# From Coarse to Fine-Grained Parcellation of the Cortical Surface Using a Fiber-Bundle Atlas

**DOI:** 10.3389/fninf.2020.00032

**Published:** 2020-09-10

**Authors:** Narciso López-López, Andrea Vázquez, Josselin Houenou, Cyril Poupon, Jean-François Mangin, Susana Ladra, Pamela Guevara

**Affiliations:** ^1^Faculty of Engineering, Universidad de Concepción, Concepción, Chile; ^2^Universidade da Coruña, CITIC, Department of Computer Science and Information Technologies, A Coruña, Spain; ^3^Université Paris-Saclay, CEA, CNRS, Baobab, Neurospin, Gif-sur-Yvette, France; ^4^INSERM U955 Unit, Mondor Institute for Biomedical Research, Team 15 “Translational Psychiatry”, Paris, France; ^5^Fondation Fondamental, Paris, France; ^6^AP-HP, Department of Psychiatry and Addictology, School of Medicine, Mondor University Hospitals, DHU PePsy, Paris, France

**Keywords:** parcellation, white matter, connectivity, fiber bundle, tractography, cortex

## Abstract

In this article, we present a hybrid method to create fine-grained parcellations of the cortical surface, from a coarse-grained parcellation according to an anatomical atlas, based on cortico-cortical connectivity. The connectivity information is obtained from segmented superficial and deep white matter bundles, according to bundle atlases, instead of the whole tractography. Thus, a direct matching between the fiber bundles and the cortical regions is obtained, avoiding the problem of finding the correspondence of the cortical parcels among subjects. Generating parcels from segmented fiber bundles can provide a good representation of the human brain connectome since they are based on bundle atlases that contain the most reproducible short and long connections found on a population of subjects. The method first processes the tractography of each subject and extracts the bundles of the atlas, based on a segmentation algorithm. Next, the intersection between the fiber bundles and the cortical mesh is calculated, to define the initial and final intersection points of each fiber. A fiber filtering is then applied to eliminate misclassified fibers, based on the anatomical definition of each bundle and the labels of Desikan-Killiany anatomical parcellation. A parcellation algorithm is then performed to create a subdivision of the anatomical regions of the cortex, which is reproducible across subjects. This step resolves the overlapping of the fiber bundle extremities over the cortical mesh within each anatomical region. For the analysis, the density of the connections and the degree of overlapping, is considered and represented with a graph. One of our parcellations, an atlas composed of 160 parcels, achieves a reproducibility across subjects of ≈0.74, based on the average Dice's coefficient between subject's connectivity matrices, rather than ≈0.73 obtained for a macro anatomical parcellation of 150 parcels. Moreover, we compared two of our parcellations with state-of-the-art atlases, finding a degree of similarity with dMRI, functional, anatomical, and multi-modal atlases. The higher similarity was found for our parcellation composed of 185 sub-parcels with another parcellation based on dMRI data from the same database, but created with a different approach, leading to 130 parcels in common based on a Dice's coefficient ≥0.5.

## 1. Introduction

The human connectome is of special interest in understanding the brain structure and function (Toga et al., 2012). The structural connectome is composed of two basic elements, the somas (nodes) and the axons (edges) that exist between them, formed by white matter (WM) tracts (Sporns et al., 2005; Hagmann et al., 2007; Bullmore and Sporns, 2009). Magnetic Resonance Imaging (MRI) provides *in-vivo* techniques to study the human brain. MRI modalities include diffusion-weighted MRI (dMRI) that estimates the WM tracts of the brain (Mori and Barker, 1999), structural MRI (sMRI) which focuses on brain anatomy (Haacke et al., 1999), and functional MRI (fMRI) that estimates brain function (Huettel et al., 2004; Van Den Heuvel and Pol, 2010). dMRI allows researchers and clinicians to non-invasively study and *in-vivo* how white matter is organized in the brain giving details of its connectivity and structure (Le Bihan et al., 2001). It is based on measurements of the movement of hydrogen atoms present in water molecules of biological tissues. Tractography algorithms reconstruct an estimate of the main WM tracts of the entire brain based on dMRI information (Basser et al., 2000; Zhang et al., 2014). The generated datasets represent an estimation of the main WM pathways, in the format of 3D polylines, also called *fibers*, even though they do not represent real neural fibers (Mori and van Zijl, 2002; Perrin et al., 2005). This technique is indirect, and relies on models and inference, but allows a whole-brain exploration of WM structure in living humans, on large populations of subjects.

The structural networks of the human cerebral cortex have not yet been comprehensively mapped (Sporns et al., 2005; Hagmann et al., 2010; Toga et al., 2012). The brain's structural and functional systems have features of complex networks, such as “small-world” topology, highly connected hubs and modularity, at the whole human brain scale (Bullmore and Sporns, 2009). The study of brain connectivity, taking into account its function and structure, can be performed based on a cortex parcellation, which is the cortical division of the brain into macroscopic regions (de Reus and Van den Heuvel, 2013). A parcellation may be based on resting state fMRI (rs-fMRI) (Schaefer et al., 2017), anatomical structure (Destrieux et al., 2010), dMRI (Lefranc et al., 2016), or cytoarchitecture. Architectonic and other template-based atlases have been created (Tzourio-Mazoyer et al., 2002; Desikan et al., 2006), but may not reflect the individual variations in regional functional boundaries. Data-driven parcellations can overcome this limitation through a better definition of individual cortical regions (Sotiropoulos and Zalesky, 2017). Parcellation atlases can be constructed using information from multiple modalities, and several scales. For example, for a given population, information from cortical folding, myelin content, resting-state, and task-based fMRI was integrated to create a functionally relevant parcellation (Glasser et al., 2016). However, individual variability and the limitations of each modality make the application of those methods very difficult. Here, we focus on the development of a method for the tractography-based parcellation (TBP) of the cortical surface. The method could be posteriorly integrated to multimodal parcellation frameworks (Parisot et al., 2017).

Connectivity-based methods use tractography information to find regions with common connectivity patterns between the cortical voxels, or cortical surface mesh vertices, that compose each region. All the methods have to deal with the high inter-subject variability, especially in the brain cortex and superficial white matter (SWM). Therefore, to reduce the complexity of the problem, some methods are focused or have been tested on a few brain regions, or have used an anatomical parcellation for initial regions (Anwander et al., 2006; Klein et al., 2007; Guevara et al., 2008; Perrin et al., 2008; Roca et al., 2010; Li et al., 2017). In general, the similarity between the connectivity profiles of the voxels (or vertices) is estimated using some similarity measure and then, a method is applied to regroup elements with common connectivity patterns. Some methods have been proposed to perform an analysis over the whole-brain cortex (de Schotten et al., 2014; Moreno-Dominguez et al., 2014; Parisot et al., 2015; Lefranc et al., 2016; O'Muircheartaigh and Jbabdi, 2017). This kind of approach, in general, calculates the whole connectivity profile of each seed node (image voxels or mesh vertices), followed by the computation of a connectivity matrix and clustering of the nodes. A group of methods performs a tractography-based parcellation of the cortex using only connectivity information given by the fiber extremities (Parisot et al., 2015; Lefranc et al., 2016; Li et al., 2017), while other groups embed fiber shape information into the analysis (de Schotten et al., 2014; Moreno-Dominguez et al., 2014; O'Muircheartaigh and Jbabdi, 2017). For an inter-subject analysis, it is also necessary to find the correspondence between subjects. One strategy is to create the parcellation taking into account the main connections present in the population of subjects (Schiffler et al., 2017). Another approach is to detect individual connectivity patterns, or even parcels, from the tractography of each subject and to then find consistent parcels among the population of subjects (Moreno-Dominguez et al., 2014; Lefranc et al., 2016; Li et al., 2017). Furthermore, due to the high complexity and the huge size of connectivity data, all the methods use a dimension reduction criterion. The difficulties mentioned above, among others, make the parcellation of the human brain cortex a complicated and unachievable task. In the following, we briefly describe some methods to provide an insight into the complexity of the solution implementation.

An interesting approach of whole-brain TBP is based on hierarchical clustering (Moreno-Dominguez et al., 2014). The method selects GM/WM interface voxels as seeds and generates probabilistic tractography from them. For each seed voxel a tractogram is obtained (visitation map). Hierarchical clustering is applied over the tractograms using a non-centered variant of the Pearson's correlation coefficient as a similarity measure. The resulting dendrogram is post-processed to reduce the number of branchings. Next, a leaf-matching is iteratively applied to the two tractograms with the highest similarity, to find correspondence across subjects. Even though the method is promising, the different parameters were difficult to adjust and no perfect match was found. Lefranc et al. (2016) apply a watershed to the connectivity profiles averaged from all the subjects of a gyrus (patch) in order to split the cortical surface into catchment basins (Roca et al., 2009). A set of regions of interest strongly connected to the gyrus across subjects is then identified, and a joint patch connectivity matrix across subjects is calculated. Finally, to construct the final cortex parcellation, each gyrus is clustered using the classical k-medoids algorithm applied to the distance matrix. The method removes a large part of the connectivity data by filtering, however, a good reproducibility among subjects was obtained. Another interesting example is the work proposed by O'Muircheartaigh and Jbabdi (2017). The method first calculates connectivity matrices from cortical vertices and subcortical voxels to the rest of the brain, based on probabilistic tractography. Then, creates an average matrix across the subjects and applies independent component analysis (ICA) to provide a group-average connectivity matrix. The dimensionality of this matrix is incrementally reduced in tractography space using principal component analysis (PCA) on subsets of the matrix. A post-processing is applied to obtain a hard parcellation of the cortex, without a straightforward mapping to tractography and gray matter, due to the high cortical and connectivity variability between subjects.

A different strategy for creating a parcellation is to use a hybrid method involving the use of bundles segmented from a bundle atlas. The first proof of concept used a subset of bundles manually selected from a multi-subject SWM bundles atlas (Guevara et al., 2017b). This work processed 10 subjects, using fix parameters manually tuned for all the processing. It segmented the bundles for each subject and calculated the intersection regions of the bundles with the cortex. In case of overlapping between two regions, the parcel label of the smaller parcel prevailed over the bigger one. This very preliminary work showed the potential advantage of using labeled bundles for the cortical parcellation, with relatively good correspondence in some regions of the brain. This method was then further improved by the use of a graph representation of the overlap between regions (Silva et al., 2019). This first attempt tuned the parameters in one subject and subsequently applied them to four other subjects, giving some correspondence across the subjects. However, since no inter-subject analysis is performed for the merging of the connecting regions, the method is not applicable to a large group of subjects.

Extending this idea, we therefore propose a new hybrid method for the structural connectivity-based parcellation of the cortical surface, based on segmented bundles. Unlike most of the methods proposed in the literature, which use full tractography, we use fibers labeled into bundles, according to short and long bundle atlases. The advantage is that the correspondence of connecting regions is given in advance for the different subjects in a database. Furthermore, the generation of parcels from segmented bundles could provide a better representation of the main regions or nodes of the human brain connectome, since these were identified as the main short and long connections of the brain, represented in the atlases of bundles. The resulting parcellation will then represent a subdivision of the cortex into the regions that connect the most probable bundles. The method still experiences difficulty in clearly defining the nodes (cortex parcels), knowing that the bundles from tractography are very variable across subjects and may not exist in several subjects. This poses a big but interesting challenge. The key point of the proposed work is the automatic analysis of the density and variability of the connecting regions among subjects over the cortical mesh, so that the most probable ones are selected, merged, and homogenized. The overlapping is solved using a graph representation of the intersected regions, taking into account the degree of overlapping of their density centers, across subjects.

The method was applied to a group of 79 subjects from a HARDI database. Several quantitative and qualitative evaluations were performed. Twenty parcellations were generated, based on different sets of the three parameters of the method, and compared to evaluate the similarity between them. Furthermore, a reproducibility analysis was also performed, based on the similarity of connectivity matrices across subjects, constructed with the whole tractography.

A comparison with a macro anatomical parcellation using Dice's coefficient between subject's connectivity matrices was performed, showing a slightly better reproducibility in a resultant parcellation generated with the proposed method. Moreover, other comparisons were also made with state-of-the-art parcellations based on different MRI modalities, finding a degree of similarity with dMRI, functional, anatomical, and multi-modal atlases. A higher similarity was found for our parcellation composed of 185 sub-parcels with another parcellation containing 239 parcels, based on dMRI data from the same database, but was created with a totally different approach. This comparison led to 130 parcels being in common based on a Dice's coefficient ≥0.5 and 75 parcels being in common with a Dice coefficient ≥0.6. Finally, complementary analyses were performed that are included in a Supplementary File.

## 2. Materials and Methods

### 2.1. Tractography Dataset

We used the ARCHI database (Schmitt et al., 2012), containing anatomical MRI and HARDI data from 79 healthy subjects with special acquisition sequences of a 3T MRI scanner with a 12-channel head coil (Siemens, Erlangen). The MRI protocol included the acquisition of a T1 image dataset using a MPRAGE sequence (160 slices, TE/TR = 2.98/2300 ms, TH = 1.10 mm, deflection angle FA = 9, TI = 900 ms; matrix = 256 × 240; voxel size = 1 × 1 × 1.1 mm; RBW = 240 Hz/pixel), a single-shell HARDI SS-EPI dataset along 60 optimized DW directions, and a field map B0, b = 1,500 *s*/*mm*^2^, (70 slices, TE = 93 ms, FA = 90, TH = 1.7 mm, TR = 14,000 ms, matrix = 128 × 128, partial Fourier factor PF = 6/8, echo spacing ES = 0.75 ms, RBW = 1,502 Hz/pixel; GRAPPA = 2, total scan time = 16 min and 46 s). The database has the affine transformation matrices to convert the data between the spaces T1, T2 (diffusion space), and Talairach. By using the software BrainVISA/Connectomist-2.0 (Duclap et al., 2012), all data were pre-processed. The main artifacts, such as noise, susceptibility effects, geometric distortions, and eddy currents were corrected. Further, defective slices were removed. The analytic Q-ball model was also computed (Descoteaux et al., 2007). Whole-brain streamline deterministic tractography was calculated, using a T1-based brain propagation mask (Guevara et al., 2011a), with one seed per voxel at T1 resolution, a maximum curvature angle of 30°, and a tracking step of 0.2 mm. Resulting tractography datasets contain about 1 million fibers per subject.

### 2.2. Approach

**Figure 1:**
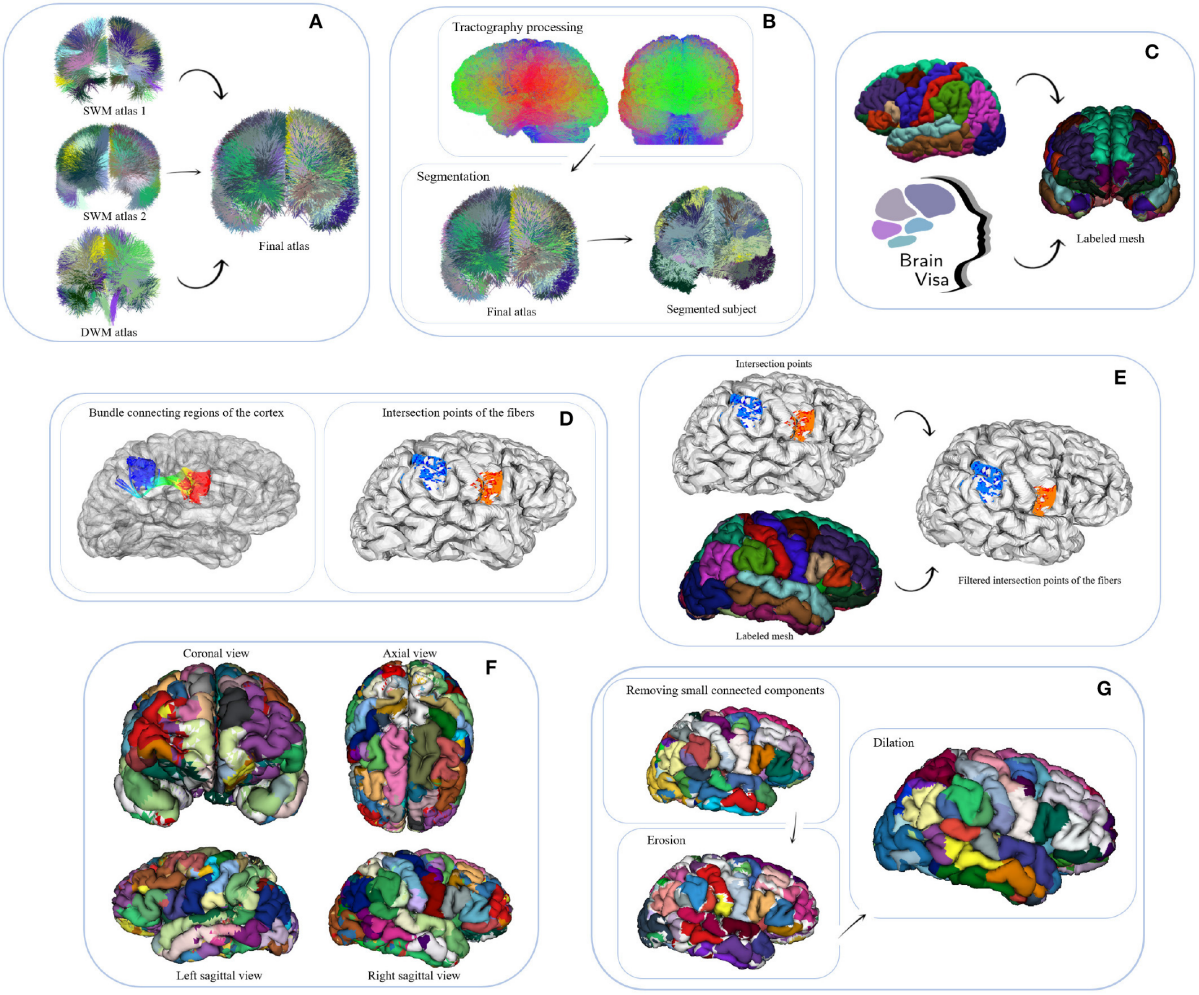
Schematic of the parcellation method. **(A)** Pre-processing: fusion of atlases. The bundles of a long and two short WM bundle atlases are fused into a final fiber bundle atlas. **(B)** Step 1: fiber bundle segmentation. A segmentation algorithm is applied to classify the fibers of each subject's tractography with respect to the final fiber bundle atlas. **(C)** Step 2: Extraction of meshes and labels. By using FreeSurfer and then BrainVISA software, the cortical meshes and their corresponding labels, according to *Desikan-Killiany* atlas, are obtained. **(D)** Step 3: intersection of the fibers with the mesh. This step intersects the fibers of each subject with its cortical mesh, obtaining the initial and final triangles intersected by each fiber bundle. **(E)** Step 4: fiber filtering. The fiber bundles are filtered according to their anatomical definition, in order to discard the fibers connecting the wrong region. This algorithm first obtains the label of each intersected triangle, then removes misclassified fibers and then performs a fiber alignment according to the corresponding atlas bundle. **(F)** Step 5: cortex parcellation. This is the main step of the parcellation method. The objective is to subdivide each region (anatomical parcel) into sub-parcels based on probabilistic structural connectivity information, derivated from a set of segmented fiber bundles. The algorithm is divided into four sub-steps: (1) creating preliminary sub-parcels, (2) calculating probability maps, (3) processing sub-parcels, and (4) merging of candidate sub-parcels. Finally, a *hard parcellation* is obtained with the most probable label for each triangle of the cortical mesh. **(G)** Step 6: sub-parcel post-processing. To get more homogeneous parcels, the small connected components are eliminated, followed by a closing of the parcels over the cortex.

We propose a hybrid method for the creation of fine-grained parcellations of the cortical surface, from a coarse-grained parcellation according to an anatomical atlas, based on structural connectivity information, given by segmented bundles for a population of subjects. The bundle segmentation is based on atlas bundles from three different atlases. The parcellation method receives as an input tractography and the labeled mesh of each subject, the fused bundle atlas with selected superficial and deep white matter bundles. The method returns an average parcellation atlas for the input dataset, which consists of a subdivision of the anatomical parcels (gyri) of Desikan-Killiany atlas, based on the most stable connectivity-based sub-parcels across the subjects. The data consists of the labels associated with each cortical mesh vertex. Note that the cortical meshes used, based on Freesurfer processing output, contain the same number of triangles and vertices in all the subjects. For all the subjects, corresponding triangles will represent the same anatomical region, but with local differences, according to the morphology of each subject. To create the final parcellation, the method uses the probability and density information of the sub-parcels from all the subjects. An intermediate output of the method is therefore the probabilistic representation of each sub-parcel. Figure 1 shows a schematic of the parcellation method. The selection of the final bundle atlas is performed as a pre-processing stage (A). Next, the method is composed of six steps: (B) fiber bundle segmentation, (C) extraction of meshes and labels, (D) intersection of the fibers with the mesh, (E) fiber filtering, (F) cortex parcellation, and (G) sub-parcel post-processing.

#### 2.2.1. Pre-processing: Fusion of Atlases

This pre-processing aims to create a fused atlas of white matter bundles, containing the main WM connections across subjects and, consequently, to create a more complete parcellation of the cortex (see Figure 1A). We used two atlases of superficial white matter (SWM) and one atlas of deep white matter (DWM). The first SWM atlas, *swm_atlas_1*, is composed of 50 bundles in both hemispheres, with a total of 7,857 fibers (Guevara et al., 2017a). The second SWM atlas *swm_atlas_2*, has 44,345 fibers and is made up of 44 bundles in the left hemisphere and 49 bundles in the right hemisphere (Román et al., 2017). Finally, the DWM atlas contains 18 bundles per hemisphere, corresponding to 11,755 fibers (Guevara et al., 2012). Those atlases were created using the ARCHI database, representing the most reproducible bundles across subjects (see Figure S2).

The bundles from both SWM atlases are labeled following the same naming convention, based on the anatomical regions of the *Desikan-Killiany* atlas (Desikan et al., 2006). The name contains *lh* or *rh* to denote the left or right hemisphere, followed by the name of the two regions connected by the bundle, according to the abbreviation of the region (see Table S3). Finally, a correlative number is added to indicate the index, as many bundles can connect the same two anatomical regions in an atlas. For example, a bundle connecting the post-central and pre-central gyri of the left hemisphere is called: *lh_PoC-PrC_0*, where 0 is the index given by the atlas. The *swm_atlas_1* contains only bundles connecting two different anatomical regions (gyri), while the *swm_atlas_2* also contains bundles connecting different areas of an anatomical region. On the other side, the DWM atlas labels the bundles according to an abbreviation of their anatomical name, followed by *LEFT* or *RIGHT* to denote the hemisphere.

To fuse the atlases, we first analyzed the bundles that are very similar in both SWM atlases, connecting the same regions. In general, in the case of a high similarity between bundles from both atlases, we selected the most compact bundle. After a visual comparison of both atlases, the bundles of the *swm_atlas_1* are better defined in their ends, and therefore are more suitable for performing a cortical parcellation. Some bundles with high similarity in both SWM atlases are shown in Figure S1. All the bundles of the *swm_atlas_1* were therefore selected (see the first row of Figure S2). Next, 27 bundles in the left hemisphere and 34 in the right hemisphere for the *swm_atlas_2* were selected, as shown in the second row of Figure S2. Most of the selected bundles of the *swm_atlas_2* connect different areas within an anatomical region.

Respecting the DWM atlas, we first discarded the Corticospinal Tract, Fornix, and Thalamix Radiations, as those bundles do not represent cortico-cortical connections. We also discarded the Corpus Callosum as it is a very large bundle that would not be very informative for the definition of subdivisions of the anatomical regions. The selected bundles are: Arcuate fasciculus, with its anterior and posterior portions (*AR, AR_ANT, AR_POST*), Cingulum (*CG*), Inferior Fronto-Occipital (*IFO*), Inferior Longitudinal (*IL*), and Uncinate (*UN*) bundles (see the third row of Figure S2). These bundles cover the cortical regions that the two SWM atlases do not cover, achieving a complete coverage of the cortex. The fused atlas is in MNI space and contains a total of 179 bundles, distributed in 86 bundles in the left hemisphere (see Table S1) and 93 bundles in the right hemisphere (see Table S2), as we see in Figure S3 (first row).

Finally, the centroid of each atlas bundle is calculated as the mean of the corresponding points of all the fibers in a bundle, to later align the segmented fibers.

#### 2.2.2. Step 1: Fiber Bundle Segmentation

This step performs the segmentation of white matter bundles for each subject (see Figure 1B). Segmenting the fibers provides direct correspondence of the bundles and the connected cortical regions across the subjects. The segmentation algorithm (Vázquez et al., 2019) is a parallel version of the algorithm proposed in Guevara et al. (2012). It classifies the fibers of a subject's tractography based on a multi-subject WM bundle atlas. It calculates the maximum Euclidean distance between corresponding points of each subject fiber and each atlas fiber. A subject's fiber is labeled with the closest bundle, if the distance does not exceed the maximum threshold defined for the bundle. The algorithm returns the subject's fibers that were correctly classified, labeled with the corresponding bundle name. Figure S3 shows the final atlas of white matter bundles as well as a subject segmented with the atlas.

To perform the segmentation, the tractography datasets are resampled with 21 equidistant points, since it is a sufficient number to perform an analysis of the similarity between fibers, as used in others works (Guevara et al., 2011b, 2012). Before the calculation, the tractographies are transformed to the MNI space.

This algorithm receives as an input the tractography of a subject, resampled with 21 equidistant points in MNI space, the fused bundle atlas, and the distance thresholds to be used for each bundle, defined for each original atlas (Guevara et al., 2012, 2017a; Román et al., 2017). It returns the segmented bundles for each subject, according to the labeling of the atlas bundles.

#### 2.2.3. Step 2: Extraction of Meshes and Labels

This step aims to obtain the meshes of the cortical surface and the labels of the anatomical regions given by the *Desikan-Killiany* atlas, as shown in Figure 1C. First, FreeSurfer software (Fischl, 2012) is employed to calculate the cortical surfaces for each subject. By using this software, a direct correspondence between the cortical surface mesh of the subjects is obtained, since the number of vertices is the same for all of them, changing only their 3D coordinates in the mesh according to the individual morphology. For the labeling of the cortical surface, FreeSurfer uses the *Desikan-Killiany* (*DK*) atlas, which consists of 35 regions per hemisphere (Desikan et al., 2006). Each region in the atlas (see Table S3) has associated a label (integer number). The labeling therefore consists of assigning each vertex of the mesh to the label of the region that it corresponds to. Next, BrainVISA software (Cointepas et al., 2010) was used to apply the pipeline that converts the formats and transforms the mesh to the subject's T1 space. It provides the mesh file with 81,924 vertices per subject and a file with the vertex labels. This step receives as input the NIFTI T1 image of each subject. The output is the cortical mesh and the labels according to the *DK* atlas, associated with each subject.

#### 2.2.4. Step 3: Intersection of the Fibers With the Mesh

This step calculates the intersection of the fibers with the cortical mesh (Silva et al., 2019). The Möller-Trumbore algorithm (Möller and Trumbore, 2005) is used to determine whether the end of a fiber intersects a mesh triangle. For each end of the fiber, the algorithm selects the nearest triangle. Finally, only those fibers whose intersections at both ends were correctly identified are used. The algorithm returns the set of initial and final intersection points for each fiber. Figure 1D illustrates a bundle and its intersection points over the cortical mesh. In this step, the algorithm receives as an input the cortical mesh and the segmented fiber bundles of each subject in T1 space. It returns for each subject and each bundle, the indices of the intersected triangles by each fiber of the bundle, at both bundle ends.

#### 2.2.5. Step 4: Fiber Filtering

This step filters out the fibers that do not connect the anatomical regions that should be connected, following the definition of the bundle to which they belong to (see Figure 1E). The algorithm receives as input the fiber intersection information (intersected triangles) and the label (cortical region) of each mesh vertex. This step returns as output the filtered fibers, i.e., those that intersect exactly within the corresponding anatomical parcel (gyri) so that all fibers that do not belong to that anatomical parcel are removed. Specifically, the filtering algorithm consists of three sub-steps.

##### 2.2.5.1. Sub-step 1: obtaining the fiber labels

**Figure 2:**
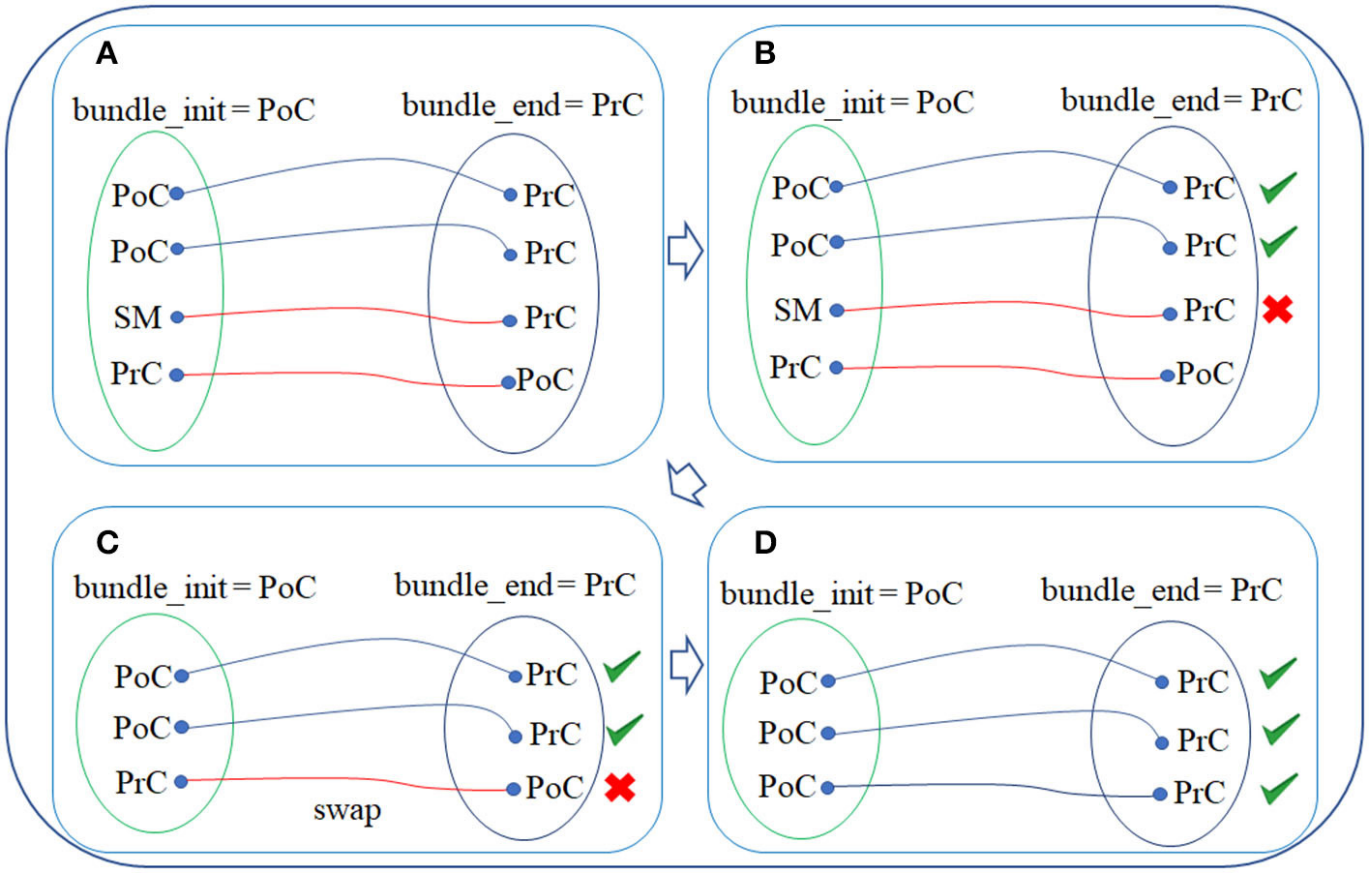
WM fiber filtering for a bundle. **(A)** Sub-step 1: obtaining the fiber labels. The labels of the triangle vertices that are intersected by the start and end of each fiber are obtained. **(B)** Sub-step 2: removing of misclassified fibers. Fibers that were misclassified by respect to the bundle anatomical definition are discarded. **(C)** Sub-step 3: fiber alignment. The fibers that are stored in the inverse direction, by respect to the atlas bundle centroid, are inversed. **(D)** Filtered final fibers for a bundle.

First, for each bundle, the labels (according to the *Desikan-Killiany* atlas) of the triangles intersected by the start and end fiber points of each fiber are obtained. Next, the names of the two regions connected by each bundle are extracted from the bundle names. For example, for bundle PoC-PrC, the initial region is PoC (post-central) and the final region is PrC (pre-central) (see Figure 2A).

##### 2.2.5.2. Sub-step 2: Removing of misclassified fibers

Each fiber is analyzed and those, in which the intersected triangle label does not correspond to the initial or final bundle regions, are removed (see Figure 2B). This processing removes the fibers that were misclassified by the fiber bundle segmentation method.

##### 2.2.5.3. Sub-step 3: Fiber alignment

The fibers on a whole-brain tractography dataset have different orientations and are stored in the direction they were tracked. Hence, on average, half of the fibers are stored in the inverse direction. In these cases, the fiber points are swapped to align the fibers according to the atlas bundles, using the bundle centroids (see Figure 2C). Finally, the filtered fibers for each bundle are obtained (see Figure 2D).

#### 2.2.6. Step 5: Cortex Parcellation

**Figure 3:**
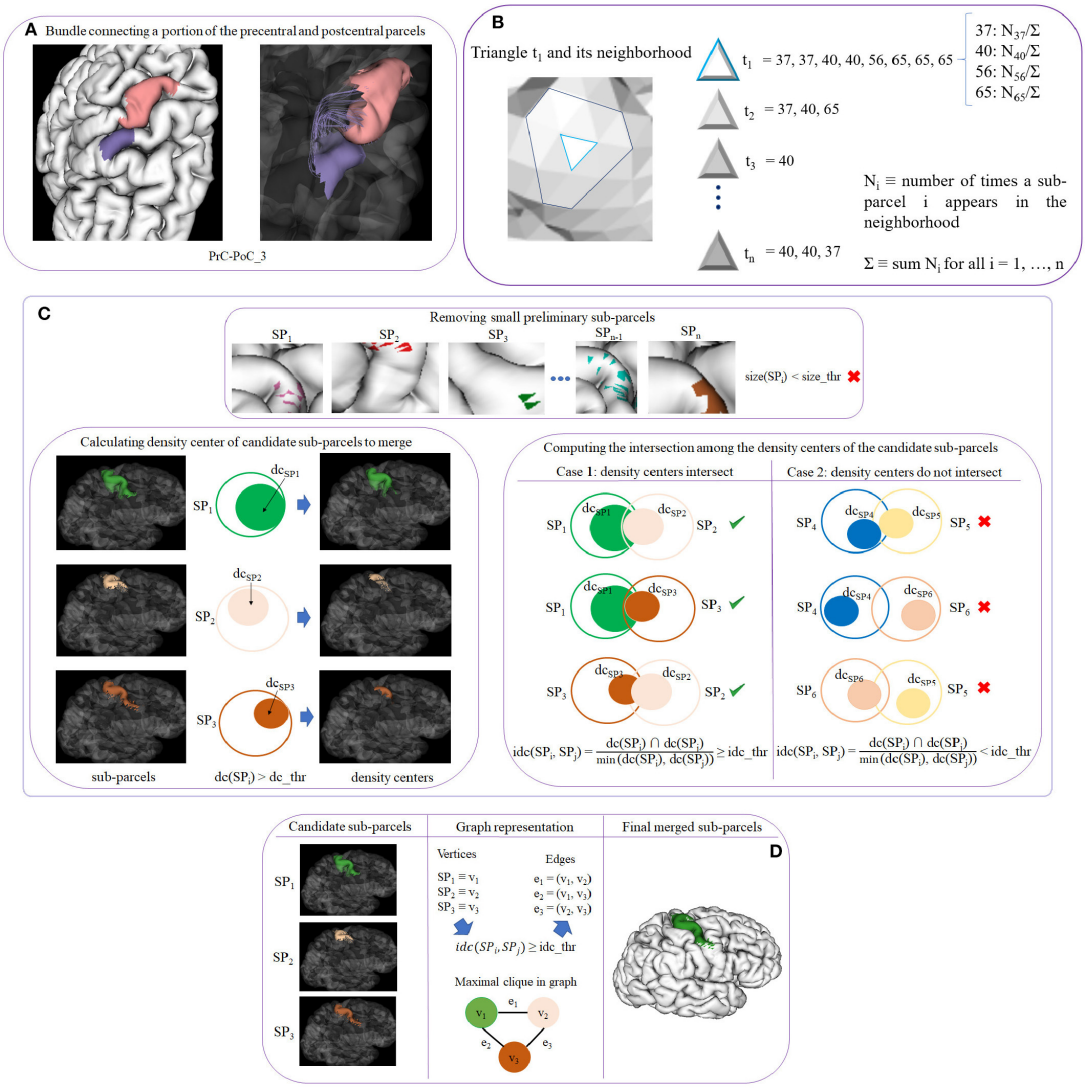
Schematics of cortex parcellation (STEP 5) sub-steps. **(A)** Sub-step 5.1: creating preliminary sub-parcels. Preliminary sub-parcels are created based on the fiber bundle intersection and the labels of each triangle. **(B)** Sub-step 5.2: calculating probability maps. The probability of each sub-parcel label in each triangle across the subjects is calculated. **(C)** Sub-step 5.3: processing preliminary sub-parcels. This step deals with the preliminary sub-parcel overlapping. First, sub-parcels that do not exceed a size threshold (*size*_*thr*) are eliminated (top image). Next, the density center (*dc*) for each sub-parcel is calculated. Preliminary sub-parcels with a *dc* greater than a threshold (*dc*_*thr*) become potential candidates to merge (bottom left image). The intersection (*idc*) of each pair of *dc* is calculated (bottom right image). **(D)** Sub-step 5.4: merging of candidate sub-parcels. In order to merge the most overlapped sub-parcels, the problem is represented by a graph, where the candidate sub-parcels are the vertices, and edges are added for the pairs of candidate sub-parcels with a high overlapping. Finally, maximal cliques are used to select the group of sub-parcels to be merged.

This is the main step of the method. It creates a fine-grained cortex parcellation, from a coarse-grained anatomical parcellation, based on the connectivity of segmented white matter bundles. The sub-parcels are probabilistic but a final hard parcellation is obtained with the most probable label for each triangle of the mesh. This algorithm receives as an input for each subject, the filtered fibers of the previous step (STEP 4), the intersection information (STEP 3), and the labeled cortical mesh (cortical mesh and vertex labels according to the *DK* atlas). It returns as an output, the mesh vertex labels for the new parcel subdivisions. As mentioned above, it also generates the probabilistic representation of each sub-parcel, which is used to generate the final parcellation. The step can be subdivided into four sub-steps (see Figure 3). Next, we explain each one of the sub-steps.

##### 2.2.6.1. Sub-step 5.1: creating preliminary sub-parcels

This sub-step creates *preliminary sub-parcels* based on the fiber bundle intersection information of each triangle. Each bundle in the atlas will define two preliminary sub-parcels, corresponding to the two extremities of the bundle. Sub-parcel names were defined following the bundle names. A label is also internally associated to identify each sub-parcel. Figure 3A shows an example of the two preliminary sub-parcels created for bundle *PrC-PoC_3* of a subject. Each anatomical parcel, given by the *Desikan-Killiany* atlas, is formed by several preliminary sub-parcels, which overlap each other, representing all the bundles that connect the region.

##### 2.2.6.2. Sub-step 5.2: calculating probability maps

This sub-step computes the probability of each sub-parcel in each triangle across the subjects. With this information, the probability maps for all the sub-parcels over the mesh are inferred, and the most probable sub-parcels for each triangle are also obtained. Let us denote *t*_*i*_ with *i* = 1, …, *n* a triangle of the mesh, and consider the neighborhood of *t*_*i*_ as all the triangles that share a vertex or and edge with *t*_*i*_. For each triangle, we count the number of times a sub-parcel appears in the neighborhood *N*_*i*_, with *i* = 1, …, *n*. To achieve this, for each triangle *t*_*i*_, a list is created with the labels of the sub-parcels that intersect the triangle or its neighborhood. A label is added for each bundle fiber intersection, for each subject. Figure 3B shows an example for a triangle *t*_1_ and its neighborhood. For instance, *t*_1_ has associated the list of sub-parcel labels that intersect the triangle and its neighbor triangles, for all the subjects. Each label has also associated the number of times the sub-parcel appears in the neighborhood (*N*_*i*_). We also calculate Σ equal to the sum of all the counts *N*_*i*_. To obtain the probability of each label in each triangle, the value *N*_*i*_ is divided by Σ. Finally, for each triangle, the list of probabilities is sorted in descending order.

##### 2.2.6.3. Sub-step 5.3: processing preliminary sub-parcels

The purpose of this step is to solve the overlap that exists between the preliminary sub-parcels within each anatomical parcel of the cortex.

First, small preliminary sub-parcels are eliminated. We denote a preliminary sub-parcel as *SP*_*i*_, with *i* = 1, …, *n* as the label of the sub-parcel, and *size*_*thr* as the threshold used to eliminate the smaller sub-parcels. The size is measured in terms of the number of triangles of the sub-parcel, as the areas of the mesh triangle are very homogeneous. The criterion of elimination *size*_*thr* is defined in terms of the percentage of triangles of the sub-parcel with respect to the corresponding anatomical region. After this processing, a set of *candidate sub-parcels* is obtained and the probability maps are recalculated according to the reduced set of sub-parcels (see Figure 3C top image).

The sub-parcels are highly overlapped, but if we look at their intersection density, we can observe that the overlapping can occur in regions with low density, or in a region of high density for one sub-parcel, but a region of low density for another sub-parcel. Indeed, the fiber intersection density is not homogeneous across the mesh surface for most of the sub-parcels. In fact, in most of the cases, only a portion of the sub-parcels present a high density. Since a merging of the sub-parcels is required to obtain a hard parcellation, we calculate the density center of each sub-parcel to perform a better analysis. The density center is defined as the area where the highest concentration of fibers exists for each triangle. We denote the sub-parcel as *SP*_*i*_ with *i* = 1, …, *n* as the label of each sub-parcel, and the density center as *dc*(*SP*_*i*_). Each triangle of a sub-parcel has associated a list with the probability of each sub-parcel present in the triangle. The density centers are then defined using a minimum probability threshold of *dc*_*thr* (density center threshold) for *dc*(*SP*_*i*_). A sub-parcel can have several density centers spread over the sub-parcel. Some examples of parcel density centers are shown in Figure 3C bottom left image. The left column displays three sub-parcels corresponding to the precentral anatomical parcel. In the middle column, we represent each sub-parcel *SP*_*i*_ as a circle of the same color and mark each density center with another filled circle inside. The third column shows the regions for *dc*(*SP*_*i*_) > *dc*_*thr*, corresponding to the density center of each sub-parcel.

Once the density centers have been calculated, we compute the intersection among them, for the candidate sub-parcels. The objective is to check if there is a significant overlap between the candidate sub-parcels, to merge them. Given all the pairs of sub-parcels, we denote the density center of the first sub-parcel as *dc*(*SP*_*i*_) and the second sub-parcel as *dc*(*SP*_*j*_), *i* and *j* being the labels of each sub-parcel, with *i, j* = 1, …, *n* and *i* < > *j*. The intersection between each sub-parcel pair is calculated based on the intersection of their density centers (triangles), following equation 1:

(1) $$\begin{array}{l} idc\left(SP_{i},SP_{j}\right)=\frac{dc\left(SP_{i}\right)\cap dc\left(SP_{j}\right)}{min\left(dc\left(SP_{i}\right),dc\left(SP_{j}\right)\right)} \end{array}$$

To define a significant intersection, we use a threshold *idc*_*thr* (intersection of density centers threshold), where *idc* ≥ *idc*_*thr* will define the sub-parcels that are candidates to merge. Figure 3C bottom right image, illustrates an example of sub-parcel intersection analysis. The first column shows a case where three sub-parcels intersect between them, considering the intersection of the three pairs of sub-parcels. The second column shows the opposite case, where no important overlaps between the sub-parcels exist, and are therefore not considered as candidates to merge. Once all the candidate sub-parcels have been obtained, the next processing step performs the merging of them.

##### 2.2.6.4. Sub-step 5.4: merging of candidate sub-parcels

In this step, the overlap between sub-parcels is analyzed using a graph representation of the sub-parcels and their intersection, to merge the parcels that are significantly intersected with each other. More specifically, the objective is to find the groups of sub-parcels that are all intersected with each other within each group. This problem can be solved using a graph representation of the sub-parcel intersections and a maximal clique algorithm.

Let *G* = (*V, E*) be an undirected graph, where each vertex *v*∈*V* represents a candidate sub-parcel. For each candidate sub-parcel pair *v*_1_ and *v*_2_ in *G*, we create an edge between them *e* = (*v*_1_, *v*_2_)∈*E* if the probability that the intersection of the density centers, *idc*(*SP*_*i*_, *SP*_*j*_) (Equation 1) is superior to the threshold *idc*_*thr*. The graph will therefore contain only the relevant intersections between sub-parcels, i. e., where the density centers present a minimum percentage of overlapping. Once *G* is created, the idea is to obtain the vertices in the graph that are all connected with each other. Therefore, we use the graph algorithm called *clique* (Karp, 1972) that aims to find subsets of vertices that are adjacent (connected), and merge them in a single sub-parcel. We use a *clique* variant called *maximal clique*, which finds a clique with the largest possible number of vertices. The problem of finding maximal cliques is that it is computationally expensive (NP-hard) (Woeginger, 2003; Sipser, 2006), however, for sparse graphs the complexity is less (Eppstein and Strash, 2011). After having calculated all the maximal cliques that are in *G*, these are sorted by size (number of vertices), in descending order. Following this order, the candidate sub-parcels of each maximal clique are merged to obtain the biggest number of fusions. This processing leads to the final sub-parcels, composed of merged candidate sub-parcels, candidate sub-parcels that were not merged, and the sub-parcels that were not candidates to merge (not included in the graph).

Figure 3D shows an example of merging for three candidate sub-parcels of the precentral anatomical parcel. In the first column, the candidate sub-parcels, denoted by *SP*_*i*_, are displayed. In the second column, a graph representation of the intersections is included, in which each sub-parcel *SP*_*i*_ is a vertex *v*_*i*_. If the *idc* (see Equation 1) between a pair of sub-parcels is superior to the threshold *idc*_*thr*, an edge is created between both vertices. The graph *G* and the maximal clique are also graphically represented. The third column shows the final sub-parcel, resulting from the merging of the three sub-parcels.

Finally, the probability maps for each triangle are recalculated. The most probable label is also determined, with the purpose of obtaining a hard parcellation (see Figure 1F).

#### 2.2.7. Step 6: Sub-Parcel Post-processing

The last step of the parcellation method deals with post-processing sub-parcels, to better define the final sub-parcels and the hard parcellation. It receives as an input the mesh vertex labels of the parcel subdivision from the previous step. The post-processing is composed of three morphological operations performed over the cortical mesh.

##### 2.2.7.1. Removing small connected components

The sub-parcels obtained in the previous step may be formed by more than one connected component. Some small components are in fact groups of a few triangles isolated from the main component. These small components are therefore removed using a graph representation of each sub-parcel. The connected components of a graph can be easily calculated (Tarjan, 1972), and then ordered by size in descending order. Next, the largest connected component is kept. For each small connected component, the second most probable label in the list containing the probability map of the corresponding triangles is selected. The neighborhood of each connected component is then analyzed to verify if a match between the second label of the triangle and its neighborhood exists. In most cases this value is appropriate, but if this is not the case, the label is removed. Figure S5 shows an example of this processing for the supramarginal (*SM*) parcel, with three sub-parcels.

##### 2.2.7.2. Sub-parcel opening

For each sub-parcel, the morphological operation called *opening* (Heijmans, 1994) is applied over the mesh, in order to eliminate isolated triangles that are scattered throughout the mesh. This operation is the result of the application of *erosion* + *dilation* operations. These two operations were therefore sequentially applied.

Figure S6 shows an example of the results after applying the post-processing, with *size*_*thr* = 0.1, *dc*_*thr* = 0.1, and *idc*_*thr* = 0.1. This hard parcellation or parcellation result, consists of 85 sub-parcels in the left hemisphere and 72 sub-parcels in the right hemisphere.

### 2.3. Parcellation Method Parameter Settings

This section provides the parcellation method configuration parameters. The parcellation method has three configurable parameters for generating a hard parcellation: *size*_*thr*, *dc*_*thr* and *idc*_*thr*. Note that all the parameters are adapted to the anatomical region and sub-parcel size, and are defined as percentages.

#### 2.3.1. Minimum Preliminary Sub-Parcel Size Threshold (*size*_*thr*)

This parameter is used to eliminate small preliminary sub-parcels that do not exceed a certain size, concerning the average size of the sub-parcels of an anatomical parcel. We visually evaluated the results with different values of *size*_*thr*, between 0.05 and 0.40. Big values, >0.25, eliminate big preliminary sub-parcels, and therefore, leave some regions in the cortex uncovered. On the other hand, values inferior to 0.1 remove only very small preliminary sub-parcels. We therefore selected a conservative value of *size*_*thr* = 0.10, which will only eliminate small sub-parcels, with a size inferior to the 10% of the average sub-parcel size on a region.

Figure S4 shows an example of *Removing of small preliminary sub-parcels* sub-step, belonging to Step 5 of the parcellation method for the precentral anatomical parcel (PrC), using *size*_*thr* = 0.10 and *size*_*thr* = 0.30.

#### 2.3.2. Preliminary Sub-Parcel Density Center Threshold (*dc*_*thr*)

This parameter determines the size of the density center (*dc*) of a preliminary sub-parcel. It defines the minimum percentage of probability of the sub-parcel in a triangle used to consider the triangle as part of the density center, and can potentially be considered for the intersection analysis. We varied its value between 0.10 and 0.30. The higher the chosen value, the smaller the density centers are, and the fewer intersections will be found when determining the intersection of the sub-parcels.

#### 2.3.3. Intersection of Density Centers Threshold (*idc*_*thr*)

This parameter defines the minimum intersection between the density centers of two sub-parcels to be considered as overlapped, and therefore, candidates to merge. This parameter is varied from 0.10 to 0.40. The lower the *idc*_*thr*, the more likely it is that the sub-parcels will merge.

## 3. Results

We implemented Steps 2 and 4 in C++11, which are also parallelized with OpenMP. The rest of the steps were performed in the Python programming language version 3.6. We executed our experiments on a computer with a 12-core Intel Core i7-8700K CPU 3.70GHz, 12MB of shared L3 cache, and 32GB of RAM, using Ubuntu 18.04.2 LTS with kernel 4.15.0-51 (64 bits). For the tests, we used the tractography datasets of the 79 subjects of the ARCHI database. We used the same database for the atlas creation and the parcellation creation to guarantee the best results. The methods are publicly available at https://github.com/andvazva/Parcellation.

Table 1 shows 20 parcellations (atlases) generated with the proposed method, resulting from different sets of parameters, with varying density center thresholds (*dc*_*thr* = 0.10, 0.15, 0.20, 0.25, 0.30) and intersection of density center thresholds (*idc*_*thr* = 0.10, 0.20, 0.30, 0.40), and a fixed value of *size*_*thr* = 0.10. The atlases are identified by a number. The table also lists the number of sub-parcels in the left hemisphere (# SP lh) and right hemisphere (# SP rh) obtained for each atlas. As *dc*_*thr* and *idc*_*thr* increase, the number of sub-parcels in both hemispheres increase, as more restrictive values are used to define a significant sub-parcel intersection, leading to an inferior number of merges.

**Table 1:** Parameters used and the number of sub-parcels obtained per hemisphere for each configuration of parameters for the cortex parcellation method.

** atlas name**	***dc*_*thr***	***idc*_*thr***	**# SP lh**	**# SP rh**
atlas 1	0.10	0.10	85	72
atlas 2	0.10	0.20	92	80
atlas 3	0.10	0.30	96	79
atlas 4	0.10	0.40	108	82
atlas 5	0.15	0.10	86	74
atlas 6	0.15	0.20	96	80
atlas 7	0.15	0.30	110	83
atlas 8	0.15	0.40	119	94
atlas 9	0.20	0.10	90	79
atlas 10	0.20	0.20	105	83
atlas 11	0.20	0.30	119	96
atlas 12	0.20	0.40	126	102
atlas 13	0.25	0.10	101	84
atlas 14	0.25	0.20	117	93
atlas 15	0.25	0.30	127	106
atlas 16	0.25	0.40	128	111
atlas 17	0.30	0.10	115	91
atlas 18	0.30	0.20	120	107
atlas 19	0.30	0.30	128	111
atlas 20	0.30	0.40	130	111

*The first column shows the name given to each atlas (parcellation result), based on a correlative number. The second and third columns list the different values for the density center (dc_thr) and the intersection of the density center (idc_thr) thresholds, for each generated atlas. Columns four and five list the number of sub-parcels obtained for the left (#SP lh) and right (# SP rh) hemispheres, respectively*.

A first observation is the asymmetry in the number of sub-parcels between both hemispheres, with more sub-parcels in the left hemisphere for all the parcellations. A similar result was found in the parcellation created by Lefranc et al. (2016), based on the same database, with 126 parcels for the left hemisphere and 113 parcels for the right hemisphere. This could be due to a higher variability found in the number of fibers across subjects in the left hemisphere. To gain insight on how much variability there is with respect to the population average, we calculated the coefficient of variation (CV) of the number of fibers in both hemispheres across subjects. CV measures the ratio between the standard deviation (σ) and the mean (μ), i. e. *CV* = σ/μ. The resulting CV for the left hemisphere is 0.23, while it is 0.21 for the right hemisphere. One possible cause of the asymmetry in the number of sub-parcels may be that the higher variability in the number of fibers in the left hemisphere could produce a higher variability of connections in the cortex, resulting in smaller parcels.

In the following subsections, we first perform a reproducibility analysis of the connectivity for the generated parcellations across subjects. A comparison of the generated atlases is then performed based on the similarity of their sub-parcels. Finally, a comparison is carried out with some state-of-the-art parcellations based on different modalities.

### 3.1. Reproducibility Analysis

**Figure 4:**
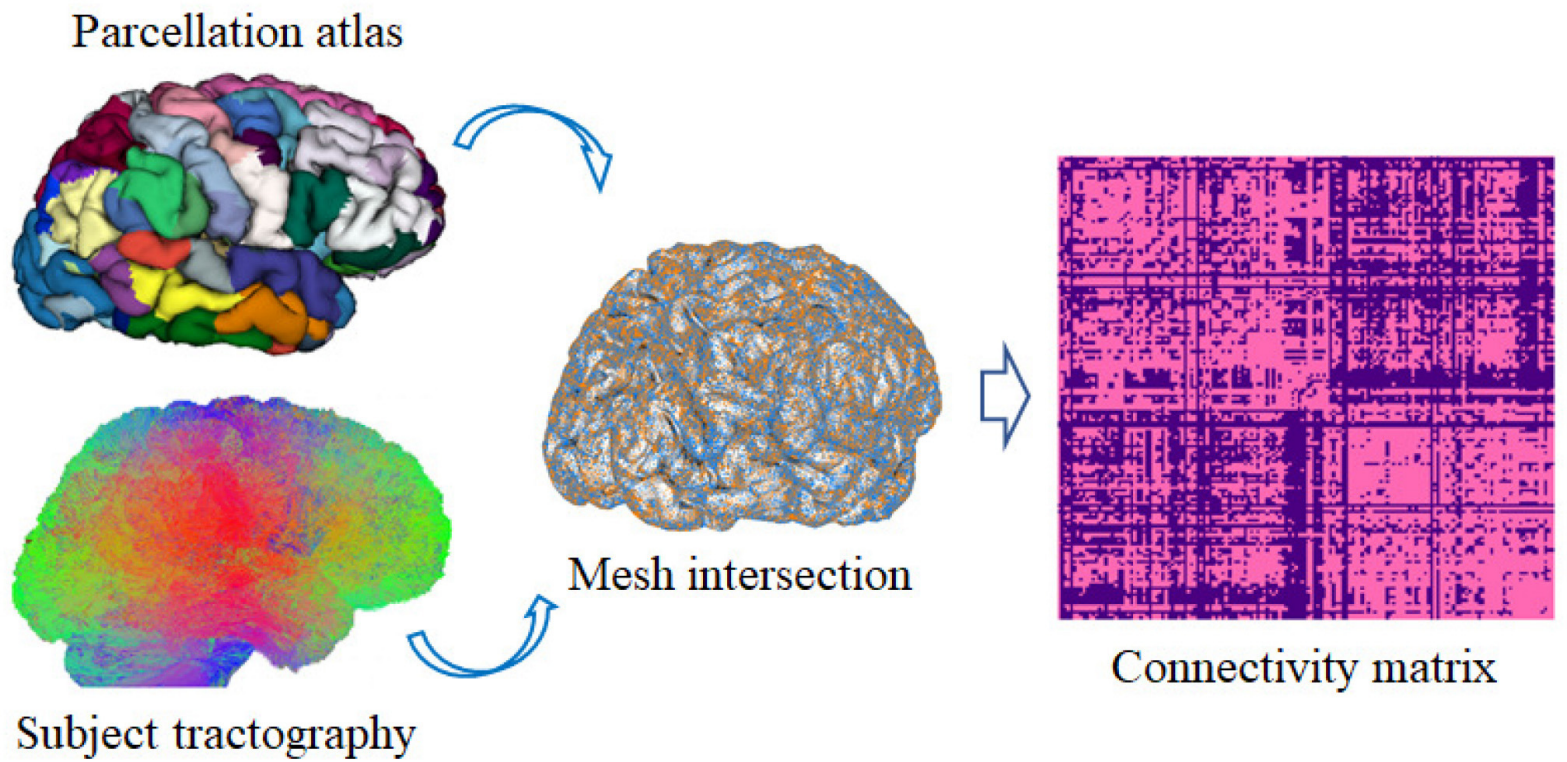
Brain connectivity analysis. First, the tractography of each subject is intersected with the subject's cortical mesh, using the generated cortical parcellation. Then, a square connectivity matrix n*n is created, where *n* is the total number of sub-parcels in the parcellation. The matrix contains a 1 where there exists a connection between the pair of corresponding sub-parcels and zero in other case.

To test the consistency of the generated parcellations across the subjects, a reproducibility analysis was performed (Arslan et al., 2018). For this purpose, for each subject, its tractography is taken and intersected with its mesh by means of the obtained parcellation. Afterwards, a binary connectivity matrix of size n*n is created, where *n* is the total number of sub-parcels belonging to the resulting parcellation. If there is a connection between two sub-parcels, it is indicated with a one, otherwise it is indicated with a zero. This procedure is shown in Figure 4. Finally, the Dice coefficient (Duda et al., 2014) is used to measure the similarity of the binary connectivity matrices across subjects for each obtained parcellation.

The Dice coefficient measures the similarity between two sets (Equation 2).

(2) $$\begin{array}{l} DSC=\frac{2|A\cap B|}{\left|A|+|B\right|} \end{array}$$

where |A| and |B| are the number of elements of sets *A* and *B*, respectively, and *DSC* is the Dice coefficient. *DSC* ranges between 0 and 1; the closer to 1, the greater the similarity between the two sets.

**Figure 5:**
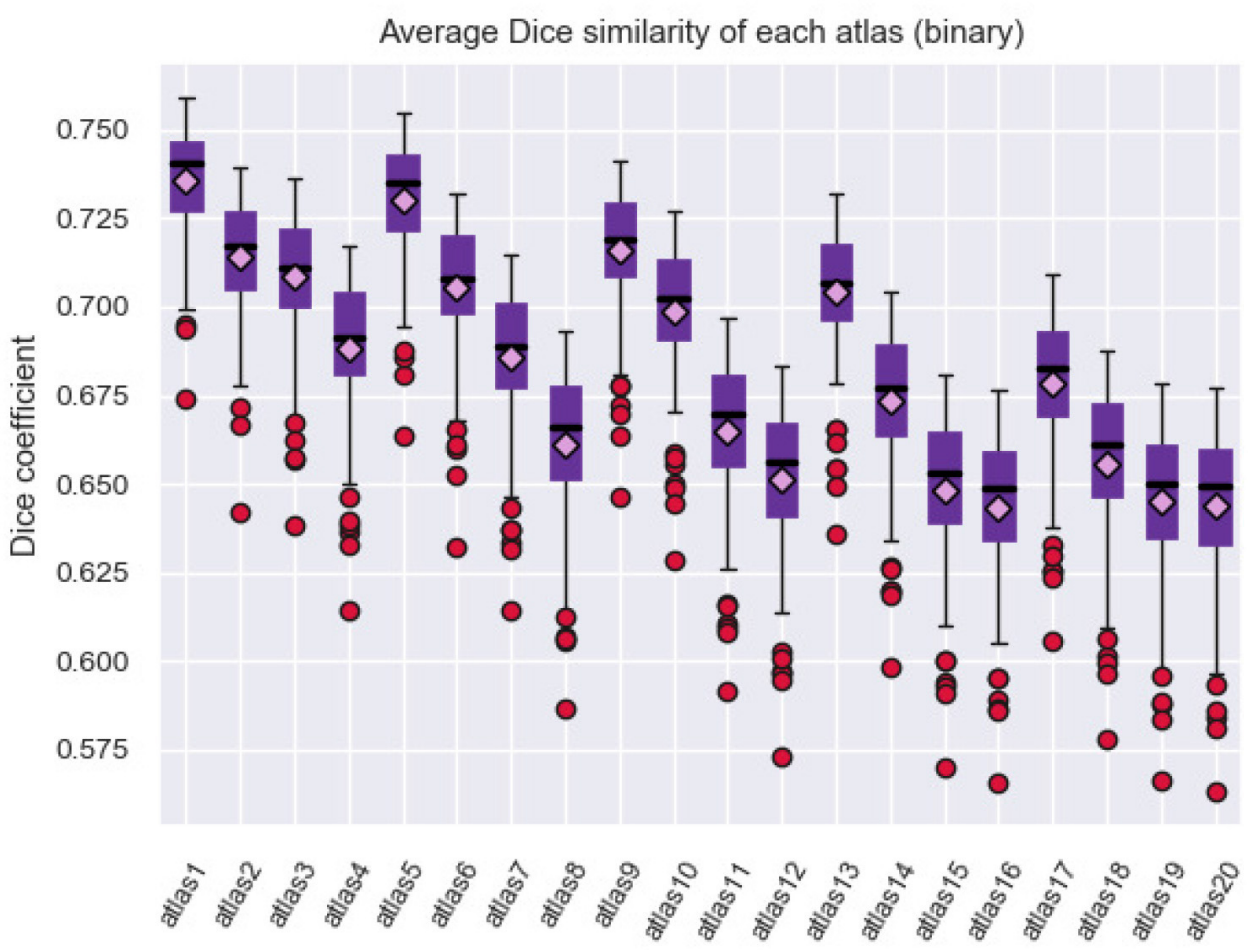
Average Dice coefficient for each parcellation (atlas) configuration, given by Table 1. The Dice coefficient was calculated between the connectivity matrices of each pair of subjects and then averaged. The results show a slight variability between the generated atlases. Due to the inter-subject variability, the similarity is smaller with a larger number of parcels.

To compute the Dice coefficient between two connectivity matrices, the *bctpy* Python library (https://github.com/aestrivex/bctpy) was used, which is an adaptation of the Matlab Brain Connectivity Toolbox for Python (Rubinov and Sporns, 2010). For each generated parcellation, *DSC* was calculated between the connectivity matrices of each pair of subjects and was then averaged (see Figure 5). A cross-validation analysis was also performed and is included in section 1.7 of the Supplementary File.

The results show that there is no great variability between the generated atlases in terms of the similarity between the connectivity matrices obtained for the different subjects. In general, the similarity decreases with the number of sub-parcels, which is expected due to the relatively bigger effect of noise and inter-subject variability, but is still good for a high number of parcels. The atlases with the least number of sub-parcels therefore have the highest similarity between the subjects, which are *atlas 1* and *atlas 5*.

Additionally, we performed some tests based on a network analysis (Bullmore and Sporns, 2009; Cohen and D'Esposito, 2016), provided in the Supplementary File. Section 1.5 describes the graph construction, while section 1.6 contains the network metrics calculation. These metrics are highly sensitive to the number of sub-parcels. For example, the results show a better small-world ω coefficient for *atlas 1* and *atlas 5*. We selected the *atlas 5* as a candidate for comparison with other state-of-the-art methods, due to its high reproducibility, and as an example of an atlas with a small number of sub-parcels.

**Figure 6:**
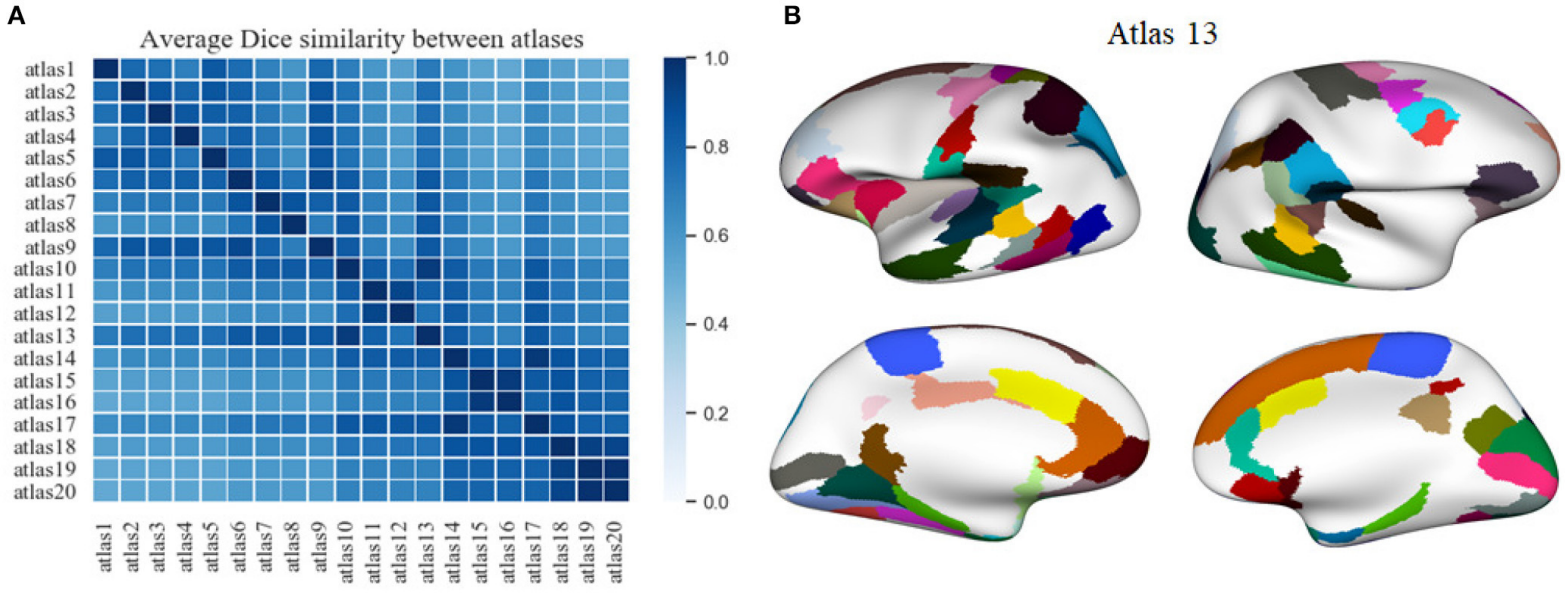
A comparison between the twenty atlases generated with the proposed method, based on different sets of the three parameters of the method. **(A)** Matrix obtained from the pairwise comparison of the sub-parcels of the different atlases, based on the average Dice coefficient (the closer to one, the more similar). The atlas most similar to the other atlases, i. e., the atlas with the higher mean Dice coefficient, is *atlas 13*. **(B)** A visualization of the sub-parcels of *atlas 13* that are in common with all the other parcellations, based on a Dice coefficient ≥0.6. The first column shows the left hemisphere with 47 sub-parcels in common, while the second column illustrates the 41 sub-parcels in common for the right hemisphere.

We applied another criterion to select a parcellation, based on the atlas that is most similar to the remaining generated atlases. This atlas is in some way the most homogeneous atlas among all the atlases generated by the parcellation method. To select the atlas, we compared between them, all the sub-parcels from the 20 generated atlases, through the construction of a similarity matrix. For each pair of atlases, we used the Dice coefficient to compare all the sub-parcels between the two atlases. To obtain the final result between two atlases, the Dice coefficient of all sub-parcels was averaged, representing the degree of similarity between the two atlases. The closer the coefficient is to one, the more similar the two atlases are. Figure 6A illustrates the similarity matrix for the 20 atlases. Finally, we selected the most reproducible atlas among the atlases, which presented the highest Dice coefficient on average, resulting in the *atlas 13*. Table S4 illustrates the number of sub-parcels per hemisphere in *atlas 5* and *atlas 13*. Table S5 shows a detailed description of the number of sub-parcels per hemisphere that have the generated atlases in common. Furthermore, Figure 6B illustrates the sub-parcels that *atlas 13* has in common with the others atlases, based on a Dice coefficient ≥0.6 (47 sub-parcels in the left hemisphere and 41 sub-parcels in the right hemisphere).

As mentioned above, we selected *atlas 5* for comparisons with state-of-the-art atlases because it is an example of an atlas with a low number of sub-parcels, and has high reproducibility. On the other hand, *atlas 13* was selected as the most similar atlas to the remaining generated atlases. *Atlas 5* contains 160 sub-parcels, while *atlas 13* contains 185 sub-parcels. Table S4 lists the differences between *atlas 5* and *atlas 13* in terms of the number of sub-parcels per each *DK* atlas region. About 70% of the sub-parcels are similar. The differences, in general, refer to a subdivision of the sub-parcels.

**Figure 7:**
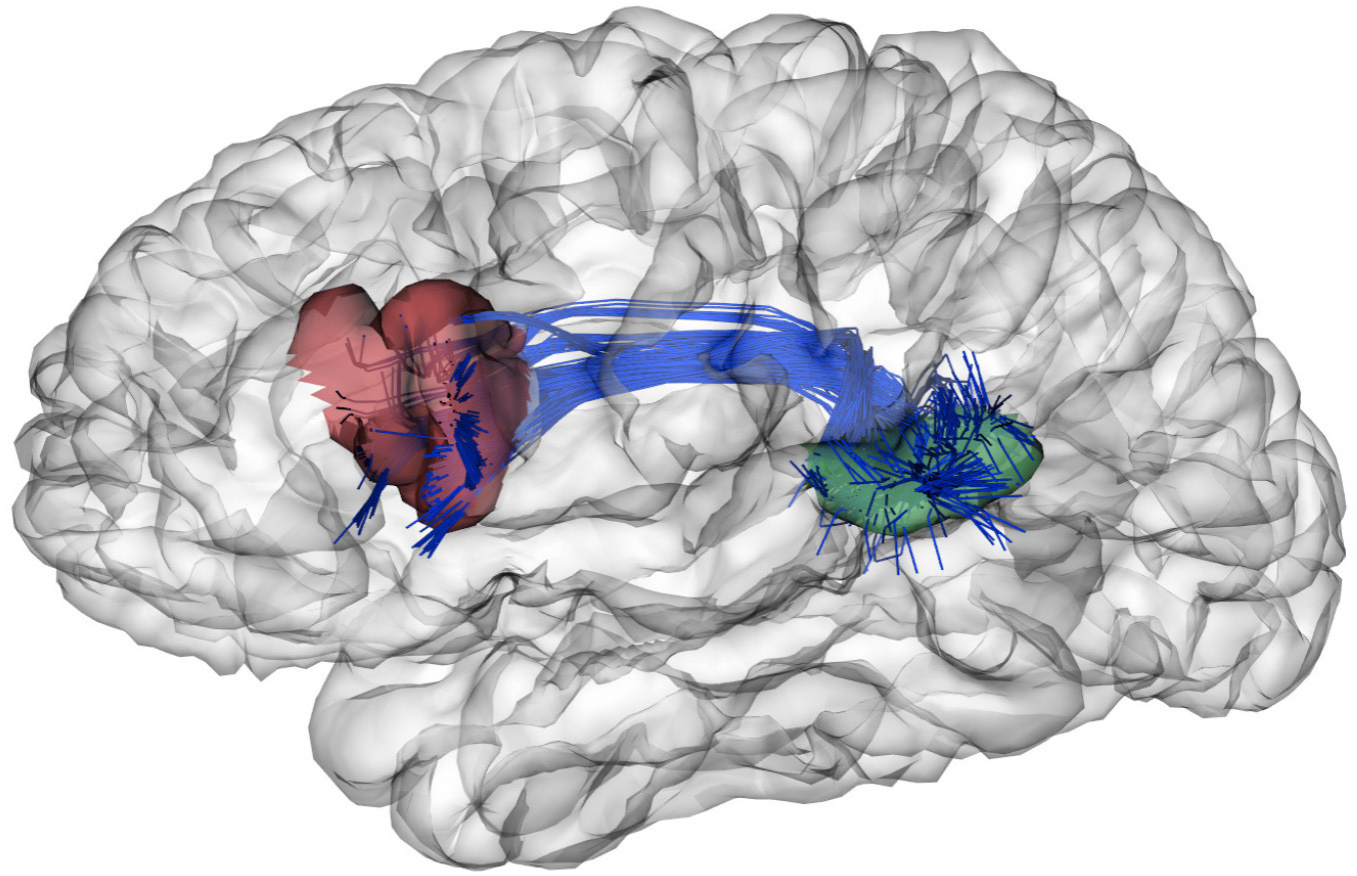
Example of two sub-parcels of *atlas 13* with biological relevance. These sub-parcels follow the most common definitions of the Broca's (Amunts and Zilles, 2012) (in red) and Wernicke's (Geschwind, 1970) (in green) areas, related to language processing. Also, the fibers connecting these sub-parcels are illustrated in blue, which correspond to the arcuate fasciculus (Catani and Mesulam, 2008).

Finally, we show an example to illustrate the biological significance of a pair of sub-parcels obtained by our parcellations. We selected the sub-parcels of *atlas 13* that better match the most common definitions of Broca's (Amunts and Zilles, 2012) and Wernicke's (Geschwind, 1970) areas, related to language processing. As illustrated in Figure 7, these regions seem to correspond to the Broca's (in red) and Wernicke's (in green) areas. Moreover, we illustrate the fibers connecting both sub-parcels, which correspond to the arcuate fasciculus, in agreement with the literature (Catani and Mesulam, 2008). In fact, this fascicle is present in the fused bundle atlas used to create the parcellations, and the segmentation of this bundle is very stable across subjects. Its connections therefore define the described sub-parcels also present in *atlas 5*. Further studies are required to validate, in detail, the biological significance of diffusion-based parcellations.

### 3.2. Comparison With State-of-the-Art Parcellations

This section provides a comparison between *atlas 5* and *atlas 13*, generated by our parcellation, with other state-of-the-art parcellations based on different modalities. For the comparisons we used *Destrieux* atlas (Destrieux et al., 2010), based on macro anatomy with 150 parcels, and *Lefranc* parcellation (Lefranc et al., 2016), based on dMRI, containing 239 parcels. Based on a multimodal approach, we used the *Brainnetome* atlas (Fan et al., 2016), which is composed of 210 cortical parcels and 36 sub-cortical parcels and *Glasser*'s atlas (Glasser et al., 2016), which contains 360 parcels, 180 per hemisphere. In addition, based on functional MRI, we used the *PrAGMATiC* atlas (Huth et al., 2016), which has 320 parcels, *Schaefer*'s atlases (Schaefer et al., 2017), consisting of several parcellations varying from 100 to 1,000 parcels, and *Yeo*'s atlas (Yeo et al., 2011), which is formed by seven or 17 networks. All the atlases are in MNI space, and are available in image format, with the exception of *Lefranc* which is available as a labeled mesh.

To be able to compare the parcellations in image format with our atlases, we first performed a mapping of the atlases to a cortical mesh. For each atlas, we labeled a cortical mesh in MNI space, by assigning the label of the closest voxel in the image atlas, to each mesh vertex. We then compared our atlases with the other parcellations, by evaluating the similarity of each sub-parcel of *atlas 5* and *atlas 13* with each parcel of the other atlases, using the Dice coefficient over the mesh triangle labels. In this case, Dice's coefficient will evaluate the degree of overlap between a pair of parcels, ranging from 0, for a total dissimilarity, to 1, for a complete similarity. Moreover, to identify the sub-parcels in *atlas 5* and *atlas 13*, we named them based on the anatomical regions connected by these sub-parcels, based on *Desikan-Killiany* atlas. For example, the parcel lh_RMF-CMF-SF_0 connects RMF with CMF and SF regions. The number (_0) denotes the index of the sub-parcel, which depends on the number of sub-parcels in an atlas with the same connections. Prefixes *lh* or *rh* refer to the left or right hemisphere. For more details, Table S3 lists the names of anatomical regions of the *Desikan-Killiany* atlas. To perform the tests, both atlases, *atlas 5* and *atlas 13*, were applied to one subject in MNI space as a representative subject (Subject 001 from the ARCHI database). The same subject was used for all tests. Any other subject transformed to MNI space could be used, because there is a correspondence between the mesh triangles of all the subjects, which have the same triangle indexes.

Tables 2, 3 contain the results of the comparison between *atlas 5* and *atlas 13* with the other atlases. For each state-of-the-art parcellation, the total number of parcels or networks that it contains, as well as the number of parcels similar to *atlas 5* and *atlas 13*, between different ranges of Dice coefficient, are indicated. In the table, the minimum Dice value considered is 0.5, while similarity values superior to 0.9 were not found.

**Table 2:** Number of similar parcels found between *atlas 5* and parcellations based on different MRI modalities.

** Parcellation name**	**≥0.5 and <0.6**	**≥0.6 and <0.7**	**≥0.7 and <0.8**	**≥0.8 and <0.9**
Brainnetome (210 parcels)	37 parcels	21 parcels	4 parcels	0 parcels
Destrieux (150 parcels)	26 parcels	8 parcels	2 parcels	1 parcel
Glasser (360 parcels)	31 parcels	7 parcels	2 parcels	0 parcels
Lefranc (239 parcels)	47 parcels	32 parcels	26 parcels	16 parcels
PrAGMATiC (320 parcels)	33 parcels	13 parcels	2 parcels	0 parcels
Schaefer (100 parcels)	26 parcels	9 parcels	2 parcels	0 parcels
Schaefer (200 parcels)	37 parcels	15 parcels	4 parcels	0 parcels
Yeo (7 networks)	0 parcels	0 parcels	0 parcels	0 parcels
Yeo (17 networks)	4 parcels	0 parcels	1 parcel	0 parcels

*For each parcellation, the total number of parcels or networks that compose it is listed, as well as the number of similar parcels based on Dice's coefficient. This coefficient ranges from 0 to 1, with 1 being total similarity. The number of similar parcels is divided into four groups, according to Dice's coefficient value*.

**Table 3:** Amount of parcels in common between *atlas 13* and other parcellations based on the Dice coefficient.

** Parcellation name**	**≥0.5 and <0.6**	**≥0.6 and <0.7**	**≥0.7 and <0.8**	**≥0.8 and <0.9**
Brainnetome (210 parcels)	40 parcels	26 parcels	5 parcels	0 parcels
Destrieux (150 parcels)	33 parcels	8 parcels	4 parcels	0 parcels
Glasser (360 parcels)	40 parcels	14 parcels	0 parcels	0 parcels
Lefranc (239 parcels)	55 parcels	35 parcels	27 parcels	13 parcels
PrAGMATiC (320 parcels)	43 parcels	19 parcels	3 parcels	0 parcels
Schaefer (100 parcels)	24 parcels	10 parcels	2 parcels	0 parcels
Schaefer (200 parcels)	31 parcels	22 parcels	4 parcels	0 parcels
Yeo (7 networks)	0 parcels	0 parcels	0 parcels	0 parcels
Yeo (17 networks)	4 parcels	0 parcels	0 parcels	0 parcels

*The first column lists the names for each parcellation and the number of parcels or networks that compose them. The other columns detail the total number of parcels in common for each Dice interval. This coefficient ranges between 0 and 1, being 1 the biggest similarity achieved*.

The *Destrieux* atlas was generated using 12 datasets and algorithms that classified each vertex in a computer-assisted manual manner and divided the cerebral cortex into 75 parcels per hemisphere, giving a total of 150 parcels. The comparison of *Destrieux* and *atlas 5*, obtained 37 similar parcels with Dice ≥0.5 in the two hemispheres. The most similar parcel is in the right hemisphere, corresponding to *G_cuneus*, located in the occipital lobe (cuneus) and corresponding to *rh_Cu-Li-MOF_0* of *atlas 5*. In the left hemisphere we have the parcels *S_suborbital* and *G&S_subcentral* corresponding to the frontal (suborbital sulcus) and parietal (subcentral gyrus) lobes. In *atlas 5* these parcels correspond to the sub-parcels *lh_MOF-LOF-LO_0* and *PoC-Ins-SM_0*, respectively. The comparison between *Destrieux* and *atlas 13* obtained 45 similar parcels with Dice ≥0.5 between both hemispheres. We highlight from the left hemisphere, the parcel *S_suborbital* of *Destrieux*, which has its equivalent in *atlas 13* of the sub-parcel *lh_MOF-IC-PrCu_0*, located in the frontal lobe (suborbital sulcus). In the right hemisphere, the parcels *G_cuneus* and *G&S_subcentral* of *Destrieux* are similar to the sub-parcels *rh_Cu-Li_0* and *rh_PoC-SM_0* of *atlas 13*, and are located in the occipital (cuneus) and parietal (subcentral gyrus) lobes, respectively.

With respect to the comparison with *Lefranc*, this parcellation has our method in common, which was based on the same database (ARCHI) and uses the regions of *Desikan-Killiany* atlas as an input. Furthermore, the method uses the whole dMRI tractography as an input. The *Lefranc* algorithm compresses the connectivity profiles of each gyrus, taking into account the inter-subject variability, and considering inter-subject high-density connectivity areas extracted using a surface-based watershed algorithm. Finally, it applies a clustering algorithm over the reduced connectivity profiles to obtain a group-wise parcellation, which consists of 239 parcels (126 in the left hemisphere and 113 in the right hemisphere). In the comparison made between *Lefranc* and *atlas 5*, we found 121 similar parcels with Dice ≥0.5. The most relevant parcel in the left hemisphere is *lh.caudalmiddlefrontal.1* which corresponds to the *CMF-PrC-RMF_1* sub-parcel in *atlas 5*, located in the frontal lobe (caudal middle frontal gyrus). The most similar parcels in the right hemisphere are *rh.inferiorparietal.3* and *rh.precuneus.2*, both belonging to the parietal lobe, specifically, the inferior parietal cortex and precuneus cortex. They correspond to the sub-parcels *IP-IT-MT_0* and *PrCu-CAC-PoCi-SF_0* in *atlas 5*, respectively. *Lefranc* and *atlas 13* have 130 parcels in common. The most similar parcels in the left hemisphere are *lh.supramarginal.2* and *lh.postcentral.3*. Both parcels are located in the parietal lobe, namely in the supramarginal and post-central gyri. The equivalent sub-parcels in *atlas 13* are *lh_SM-PrC_0* and *lh_PoC-Ins-SM_0*. In the right hemisphere, we found *rh.inferiorparietal.2* parcel, which is located in the parietal lobe (inferior parietal cortex) and is similar to sub-parcel *rh_IP-SM-PrC_0* in *atlas 13*.

The *Brainnetome* atlas relies on a multimodal approach. Multimodal information consists of diffusion MRI, functional MRI, and structural MRI data. This parcellation was based on 80 subjects of the Human Connectome Project (HCP) database and contains 210 cortical parcels (105 per hemisphere) and 36 subcortical parcels. This atlas has *atlas 5* and *atlas 13* which uses dMRI, in common, but employs probabilistic rather than deterministic tractography. Furthermore, both methods use the *Desikan-Killiany* atlas as input information. *Atlas 5* and *Brainnetome* have 62 parcels in common. We can highlight *parcel A9_46d_L* (left hemisphere) located in the frontal lobe (middle frontal gyrus), which is linked to the *atlas 5* sub-parcel called *lh_RMF-CMF-SF_0*, and is related to inhibition, social cognition, and word generation. In the right hemisphere, parcel *msOccG_R* of *Brainnetome*, located in the occipital lobe (lateral occipital cortex), is similar to sub-parcel *rh_SP-LO-MT_0* of *atlas 5*, which is related to spatial ability, shape vision, motion vision, and inhibition. We also have parcel *A23c_R* which is similar to sub-parcel *rh_PoCi-CAC-PrCu-RAC_0*, located in the limbic lobe (cingulate gyrus), and which is related to emotions, reward, and pain. Moreover, *atlas 13* and *Brainnetome* have 71 parcels in common. The three most similar parcels correspond to the left hemisphere. *Brainnetome* parcel *A9_46d_L* corresponds in *atlas 13* to the sub-parcel *lh_RMF-CMF-SF_0* (middle frontal gyrus) and is related to inhibition, social cognition, and word generation. Parcel *A40rv_L* is linked to sub-parcel *lh_SM-PrC_0* in *atlas 13* and is located in the parietal lobe (inferior parietal) and has functions related to audition, pain, grasping, and discrimination. Finally, parcel *A8dl_L* corresponds to sub-parcel *lh_CMF-Op_0* of *atlas 13*, is located in the frontal lobe (superior frontal gyrus), and is related to emotion, cognition, and memory.

*Glasser* atlas is also based on a multimodal approach. This parcellation is based on functional connectivity (resting state), microstructural architecture, functional specialization (task-fMRI), and topography information. In addition, it uses data from 449 subjects of the HCP database and generates a final parcellation which consists of 360 parcels (180 per hemisphere). We found 40 parcels in common with *atlas 5*. In the left hemisphere, sub-parcel *lh_Or-LOF-Ins-LO_0* of *atlas 5* corresponds to parcel *L_a47r_ROI* located in the frontal lobe (Orbital and Polar Frontal Cortex) of *Glasser* and is linked to relational-match, gambling, working memory, language (story and math), and face-shape recognition. Continuing in the same hemisphere, another relevant *Glasser* parcel is *L_POS1_ROI*, located in the parietal lobe (Posterior Cingulate Cortex), which is similar to sub-parcel *lh_PrCu-PH-En_0* and is related to language (story and math) and scene selection. As for the right hemisphere, the *R_V3A_ROI* parcel which is located in the occipital lobe (Dorsal Stream Visual Cortex) is equivalent to the *rh_SP-LO-MT_0* sub-parcel of *atlas 5*, which is related to retinotopic areas, gambling, and emotion. The The comparison between *Glasser* and *atlas 13* led to 54 parcels being in common. The most similar left hemisphere for *Glasser* are *L_TPOJ1_ROI* and *L_11l_ROI*, which are linked to the sub-parcels *lh_Ban-MT_0* and *LOF-LO_0* of *atlas 13*. The former is located in the Temporo-parieto-occipital junction, an area associated with faces-shapes recognition, language (story and math), audition, visual concepts, and gambling. *LOF-LO_0* parcel is in the frontal lobe (Orbital and Polar Frontal Cortex) and has the functionalities of memory and face-shape recognition. Regarding the right hemisphere, the *R_V3A_ROI* parcel of *Glasser* has its equivalent in the sub-parcel *rh_IP-IT_0* of *atlas 13*, located in the occipital lobe (Dorsal Stream Visual Cortex) and is related to gambling, emotion, and retinotopic areas.

The *PrAGMATiC* atlas is the result of using fMRI and ROI-based methods in a probabilistic and Bayesian generative model approach. The model was applied using 12 subjects, and the resulting atlas, containing 320 parcels, represents the distribution of semantically selective functional areas in the human cerebral cortex. The comparison made between *PrAGMATiC* and *atlas 5* found 48 common parcels. We highlight in the left hemisphere parcel *IPFC_L8* of *PrAGMATiC*, located in the frontal lobe (inferior prefrontal cortex), which is similar to sub-parcel *lh_Tr-Ins-SF-IT_0* of *atlas 5* and is related to violence, emotions, social, and abstract skills. Continuing in the same hemisphere, we have the parcel *IPFC_L12* located in the frontal lobe, similar to sub-parcel *lh_LOF-ST-TEM-LO_0* of *atlas 5*, which is related to abstract, tactile, and numeric skills. On the other hand, in the right hemisphere, parcel *LTC_R3* of *PrAGMATiC* is equivalent to sub-parcel *rh_Ban-IP_0* of *atlas 5* located in the temporal lobe (lateral temporal cortex) and which is related to violence, social and emotion skills. *PrAGMATiC* has 65 parcels in common with *atlas 13*. The *LTC* parcel in the left hemisphere is the most similar, located in the temporal lobe (lateral temporal) and equivalent to sub-parcel *lh_Ban-MT_0* of *atlas 13*, with associated functionalities, such as violence, emotional, and communal skills. In the right hemisphere we highlight the parcels *LTC_R3* and *SPFC_R15*, located in the temporal (lateral temporal) and frontal (superior prefrontal) lobes. The former has the functionalities of violence, social, and emotional concepts, while the latter is associated with mental, emotional, and violent concepts.

Schaefer is a parcellation based on resting-state fMRI from a database of 1,489 subjects, which uses a gradient-weighted Markov Random Field (gwMRF) model to generate the final parcellations, with 100, 200, 400, 600, 800, and 1,000 parcels. To compare the *Schaefer* atlas with our results, we chose the parcellations of 100 and 200 parcels because they are the most similar in number of parcels to *atlas 5* (160 sub-parcels) and *atlas 13* (185 sub-parcels). Starting with *Schaefer* parcellation with 100 parcels, we found 37 parcels in common with *atlas 5*. In the left hemisphere, parcel *DefaultB_IPL_1* of *Schaefer* is similar to sub-parcel *lh_IP-IT-MT_0* of our atlas and is located in the temporal lobe, while in the right hemisphere, parcel *ContB_IPL_1* is equivalent to sub-parcel *rh_SM-PrC-SP_0* and is located in the frontal lobe. Both parcels are associated to language skills (story and math). We also highlight sub-parcel *rh_RMF-LOF-SF-LO_0* of *atlas 5*, which corresponds to parcel *ContB_PFCl_1* of *Schaefer* located in the parietal lobe, and is related to the working memory.

On the other hand, the *Schaefer* parcellation of 200 parcels, has a total of 56 parcels that are in common with *atlas 5*. In the left hemisphere, parcel *Temp_Par_1* (temporal lobe) from *Schaefer* is similar to sub-parcel *lh_Ban-IT-MT_0* of our atlas, which is associated to language functionality (story and math). For the right hemisphere, parcel *TempPar_3* (temporal lobe) of *Schaefer* is similar to *rh_Ban-IP_0* sub-parcel of *atlas 5*, which is related to language (story and math), and the *VisCent_ExStr_3* parcel is similar to the *rh_LO-Or-MT-RMF_0* sub-parcel, which is located in the occipital lobe and is associated to visual areas and relational skills (matching and fixation). Moreover, the comparison between *Schaefer* parcellation *atlas 13* found 57 similar parcels. In the left hemisphere the most similar is the parcel *LimbicA_TempPole_1* located in the temporal lobe, which is related to the sub-parcel *lh_TEM-LOF-MOF-LO_0* of the *atlas 13* and has is associated to functionality of language (story and math). For the right hemisphere, *TempPar_3* (temporal lobe) parcels are equivalent to sub-parcel *rh_Ban-IP_0* of *atlas 13*, which is associated to language functionalities (story and math), and parcel *VisCent_ExStr_3* (occipital lobe) is similar to the *LO1-LO0-MT_1* sub-parcel, which is associated to relational skill functionalities (matching and fixation).

The *Yeo* atlas was based on fMRI data from 1,000 subjects. The comparison with this atlas leads to less similar parcels, since the sub-parcels of *atlas 5* and *atlas 13* are generally smaller than the 17 networks of the *Yeo* atlas. Five parcels were found to be in common with the *Yeo* atlas and *atlas 5*, and four parcels between *Yeo* atlas and *atlas 13*. We highlight sub-parcel *lh_LO1-LO0-ST-MT_1* from located in the left occipital lobe, which is related to the *V1*_*c*_ region of the *Yeo* atlas and is associated to the visual area and central vision.

**Figure 8:**
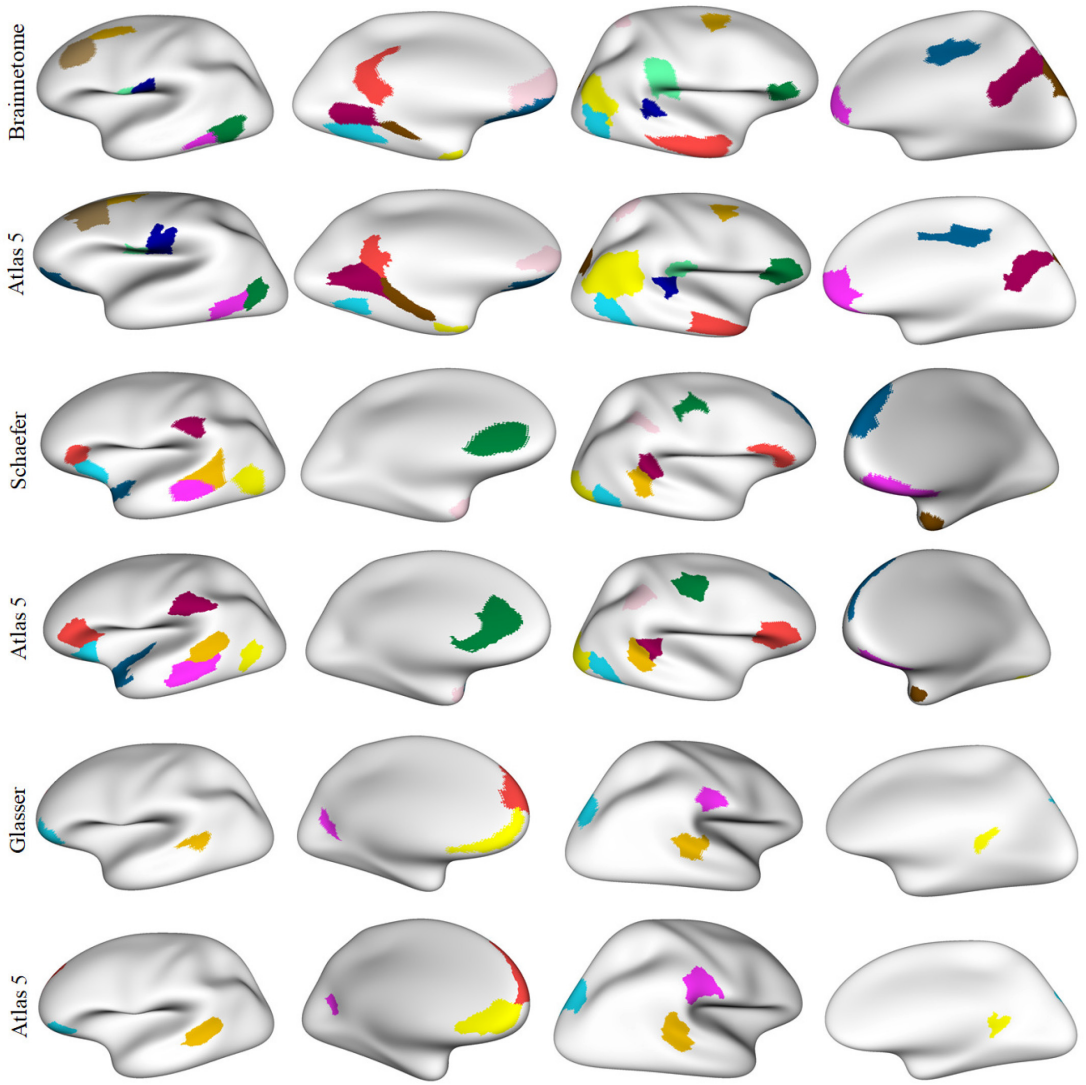
Parcels in common between *atlas 5* parcellation and some parcellations based on different MRI modalities, with Dice coefficient ≥0.6. Both hemispheres are shown for each parcellation with the inflated mesh. First and second rows: comparison with *Brainnetome* (210 cortical parcels) (Fan et al., 2016), 13 similar parcels were found in the left hemisphere and 12 in the right hemisphere. Third and fourth rows: comparison with *Schaefer* parcellation (200 parcels) (Schaefer et al., 2017), with 9 similar parcels in the left hemisphere and 10 in the right hemisphere. Fifth and sixth rows: comparison with *Glasser* parcellation (360 parcels) (Glasser et al., 2016), with 5 similar parcels in the left hemisphere and 4 in the right hemisphere. This gives a total of 25, 19, and 9 parcels in common, respectively.

**Figure 9:**
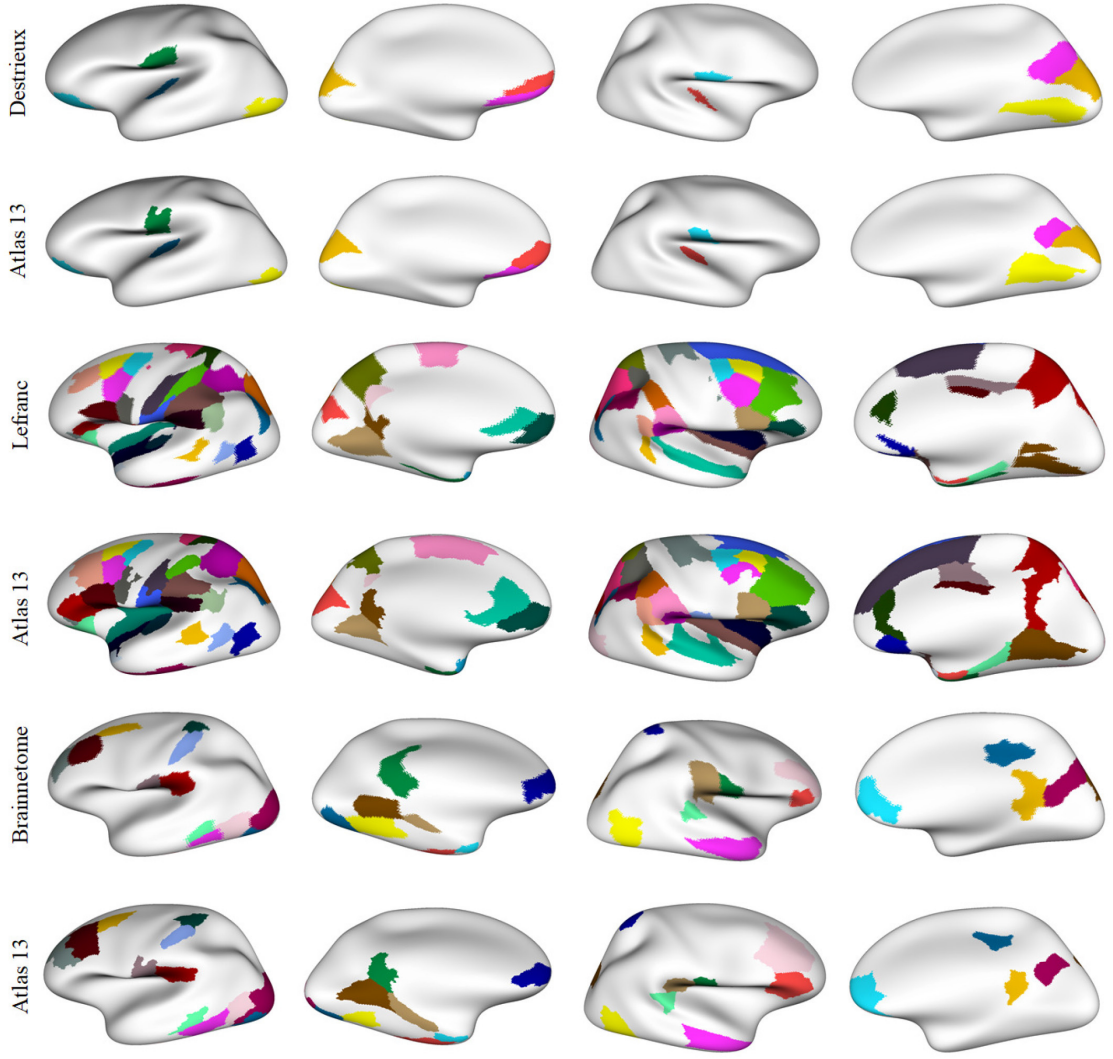
Common parcels between *atlas 13* parcellation and some parcellations from the state-of-the-art with Dice coefficient ≥0.6. Both hemispheres are shown for each parcellation with the inflated mesh. First and second rows: comparison with *Destrieux* atlas (150 parcels) (Destrieux et al., 2010), with 7 parcels in common in the left hemisphere and 5 in the right hemisphere. Third and fourth rows: comparison with *Lefranc* atlas (239 parcels) (Lefranc et al., 2016), with 40 common parcels in the left hemisphere and the 35 parcels in the right hemisphere. Fifth and sixth rows: comparison with *Brainnetome* (210 cortical parcels) (Fan et al., 2016), with 19 parcels in common in the left hemisphere and 12 parcels in the right hemisphere. This gives a total of 12, 75, and 31 similar parcels, respectively.

Figure 8 illustrates the parcels found to be similar between *atlas 5* and other parcellations, mainly based on functional information (*Brainnetome, Schaefer* and *Glasser*), considering a Dice coefficient ≥0.6. Figure 9, shows the common parcels between *atlas 13* and *Destrieux, Lefranc* and *Brainnetome* parcellations, with a Dice coefficient of ≥0.6. For more information on comparisons see section 1.9 of the Supplementary File.

The evaluation of the differences between the atlases based on diffusion MRI (*Lefranc, Brainnetome*, and our parcellations) is a difficult task. The coarse anatomical regions in which the atlases were based is the main difference. *Lefranc* and our parcellations present a high dependency on the anatomical regions of the *Desikan-Killiany* atlas (35 per hemisphere), while *Brainnetome* uses the *DK* atlas but with several regions combined (20 cortical and 4 subcortical regions per hemisphere). We can therefore compare the granularity of the *DK* regions for the different atlases, where a higher difference exists for *Brainnetome* in the combined regions. Table S6 lists the number of subdivisions of *DK* anatomical regions for all the atlases. In some cases, *Brainnetome* parcels cannot be matched with DK standard regions. In the table, an asterisk is used to indicate the *DK* anatomical parcels where *Brainnetome* performs a different subdivision of the regions and only an approximate number of subdivisions is provided. Another big difference is that *Brainnetome* has equivalent parcels in both hemispheres, while *Lefranc* and our parcellations are asymmetric, presenting more sub-parcels in the left hemisphere. This is due to the different approaches used, where the method that created *Brainnetome* was used, in addition to stability across the population, and the interhemispheric anatomical homology. Another difference is the number of total parcels, where *Lefranc* has 239, *Brainnetome* 210, *atlas 13* 185, and *atlas 5* 160 cortical sub-parcels. Depending on the application, the total granularity could therefore be the determinant for the atlas selection. Furthermore, *Lefranc* presents subdivisions in almost all the *DK* atlas regions, which is not the case for the other atlases. This could be produced by the watershed algorithm applied to the cortical surface, which may be more sensitive to local connectivity density variations. This atlas also presents a high granularity in some regions, such as the Fusiform, Lateral occipital (left), Middle temporal, Pars orbitalis, Pericalcarine, and Transverse temporal. *Brainnetome*, on this side, presents more subdivisions for the Inferior temporal, Superior frontal, and Insula regions. Finally, the four atlases present higher subdivisions for the Superior temporal, Superior frontal, Pre-central, Post-central, and Inferior temporal gyri. Furthermore, *Brainnetome* is the only atlas that has subcortical parcels (18 per hemisphere).

**Figure 10:**
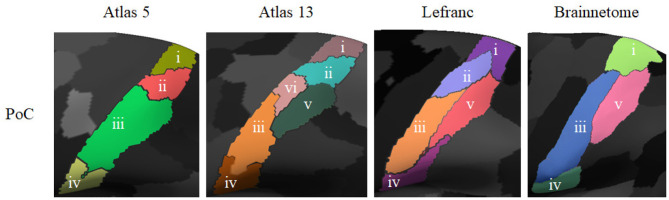
Comparison of the sub-parcels obtained by the different atlases based on dMRI for the post-central (PoC) anatomical parcel. From left to right the atlases are: *atlas 5* (four subdivisions), *atlas 13* (six subdivisions), *Lefranc* (five subdivisions), and *Brainnetome* (four subdivisions). The sub-parcels were enumerated following the best correspondence between atlases. It can be observed that sub-parcels *iii, v*, and *vi* of *atlas 13* are a subdivision of sub-parcel iii of *atlas 5*. Also sub-parcels *i* and *iv* are similar in all the atlases. Furthermore, sub-parcels *v* in *atlas 13, Lefranc*, and *Brainnetome* are very similar.

Making a comparison between the atlases based on dMRI modality, we found similarities and differences in the number of subdivisions per anatomical parcel and per hemisphere. Figure 10 shows a visual comparison between *atlas 5, atlas 13, Lefranc*, and *Brainnetome* for the post-central anatomical parcel. The sub-parcels found were enumerated according to their correspondence between the different atlases. Sub-parcels *i* and *iv* are similar for all the atlases. In addition, sub-parcel *iii* of *atlas 5* is divided into sub-parcels *iii, v*, and *vi* of *atlas 13*. On the other hand, there is a high similarity between sub-parcels *v* in *atlas 13, Lefranc*, and *Brainnetome*.

**Figure 11:**
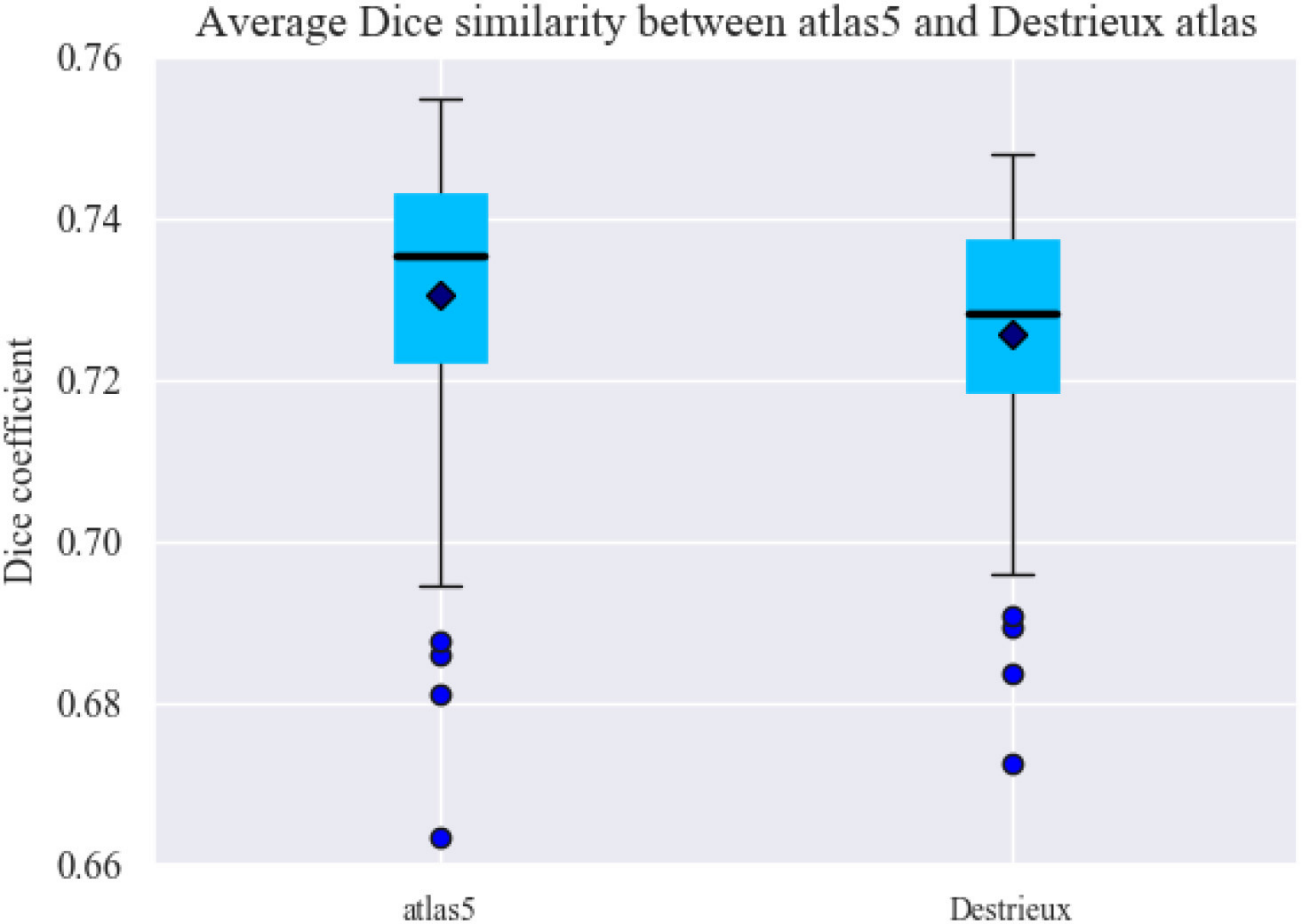
Dice coefficient for connectivity reproducibility for *atlas 5* parcellation and *Destrieux* atlas. Dice's coefficient is in the range zero to one, the closer to one the more reproducibility between subjects. *Atlas 5* composed of 160 sub-parcels is slightly better in terms of reproducibility than the *Destrieux* parcellation consisting of 150 parcels.

Finally, we compared the connectivity matrices obtained for *atlas 5* (160 sub-parcels) and the *Destrieux* atlas (150 parcels). First, the connectivity matrices of each subject for both atlases were calculated (79 subjects). The matrices were then binarized and the Dice coefficient was calculated between each pair of subjects and posteriorly averaged for each atlas, to compare the reproducibility of the connectivity matrices generated by both atlases. Figure 11 shows the results of the Dice coefficient for both parcellations. As shown, the *atlas 5* parcellation is a little more reproducible (≈0.01) than the *Destrieux* atlas, despite having 10 more parcels. In general, the higher the number of parcels, the lower the value of inter-subject reproducibility using Dice's coefficient, as subdividing the cortex into a larger number of sub-parcels will lead to more variable connectivity, due to inter-subject variability. With the obtained result, it seems that the boundaries of the sub-parcels produce a better agreement with the underlying connections.

## 4. Discussion

We propose a new hybrid method for the creation of fine-grained parcellations of the cortical surface from a coarse-grained anatomical parcellation, based on the connectivity given by a fiber-bundle atlas. Since the bundles have a correspondence between subjects, a direct match is obtained between the regions intersected by the extremities of the bundles across subjects. However, due to the overlap of cortical bundle intersections, inter-subject variability, and tractography limitations, several processing steps are applied to find consistent parcels among subjects.

The main analysis uses the probability of each sub-parcel along with the fiber density over the cortex to detect reproducible regions. The overlap between regions is also solved using a graph representation of sub-parcel density center intersections. The method has the advantage of being conceptually simple, despite the complexity of its implementation, with few parameters that represent characteristics that are also easy to understand. Therefore, parameter variation has an understandable effect on the final parcellation, in particular, in the number of sub-parcels. The results are very promising, showing an expected behavior of the method for a wide range of parameters and a high similarity between the generated atlases. Even though the final number of sub-parcels per hemisphere depends on the parameter configuration, there is a high dependence on the maximum number of sub-parcels with the bundle atlas used. This is the reason why the atlas *swm_atlas_1* (Guevara et al., 2017a) was chosen first, as it contained much more compact bundles at its extremities, leading to more candidate sub-parcels. Furthermore, the optimal number of sub-parcels and the method itself will depend on the subsequent analysis to be performed. If the objective is to analyze and compare structural connectomes, it seems convenient to use connectivity-based parcellations created from tractography data.

In general, our method leads to good inter-subject correspondence in all the created parcellations, given by a relatively high average Dice coefficient for connectivity matrices. The comparison with the *Destrieux* atlas showed a slightly better reproducibility for *atlas 5*, despite having 10 more sub-parcels. The contribution of this work is a method for the creation of a fine-grained parcellation from an anatomical coarse parcellation, based on a bundle-atlas that can be used for further analyses.

The comparisons between *atlas 5* and *atlas 13*, with some atlases based on different modalities, found a set of similar parcels, with a Dice coefficient ≥0.5. The comparison with parcellations based on fMRI provided some insight on the functions related to some sub-parcels obtained for our atlases, which in turn are associated with specific structural connections. Even though the objective of the present work is to propose a diffusion-based parcellation, the comparison shows that good correspondence is found between several sub-parcels of our atlases and parcels from other modalities. For comparison, the size of the parcel is crucial. For example, for the *Yeo* atlas with seven networks, no similar parcels were found due to the higher size of the networks. On the other hand, ≈51% of the parcels in the *Lefranc* atlas (with 239 parcels), based on dMRI, have a similar sub-parcel in *atlas 5* (with 160 sub-parcels). The comparison between the *Lefranc* atlas and *atlas 13* achieves ≈54% common parcels (130 sub-parcels) with the most similar parcellation. This result is interesting since the proposed method is based on the same database than the *Lefranc* atlas, but with a totally different approach. Despite the different number of parcels, two other parcellations have more than 30% of similarity with our atlases, which are *Brainnetome* and *Schaefer* with 100 parcels. Of course, we are not considering a perfect match between the parcels. Further analyses could be performed, in particular, to compare the different state-of-the-art parcellations, but are out of the scope for this study.

A limitation of the method may be the use of the fused fiber atlas, composed of SWM and DWM bundles, to generate the input data, instead of using the whole-brain tractography. However, all the diffusion-based methods, at some stage, perform a filtering of the data, since it is necessary to keep only the reproducible regions or connections. A concrete limitation is the maximum number of sub-parcels that could be created, which depends on the final atlas bundles. We found a total number of sub-parcels ranging from 157 to 241, which is around the number of parcels obtained by the state-of-the-art methods based on tractography, with 15–250 parcels for Moreno-Dominguez et al. (2014), 239 parcels for Lefranc et al. (2016), and 50–300 parcels for O'Muircheartaigh and Jbabdi (2017). To reach a higher number of parcels it would be necessary to add more bundles or to subdivide the current bundles. Another limitation of the method is the use of the Desikan-Killiany atlas to define the coarse granularity of the sub-parcels, instead of generating a parcellation without such limits. The entire method is also difficult to reproduce due to the use of different platforms and methods, which is not infrequent in this type of analysis. For that reason, we have created a code repository at (https://github.com/andvazva/Parcellation) with the all the codes and files necessary to apply all the processing steps, including the fused bundle atlas, and the segmentation, intersection and parcellation codes, among others. Furthermore, the resulting data will be available to the public in a data repository.

On the other hand, the use of the fused fiber bundle atlas is an advantage, since it allows for a direct correspondence between subjects, avoiding the search for such correspondence at the end of the process by employing clustering algorithms. Another positive aspect is the low execution time, where the segmentation algorithm is capable of segmenting a subject of 1,500,000 fibers in <20 s and the cortical parcellation algorithm performs the subdivision of the anatomical *DK* parcels in ~10 min. Furthermore, this algorithm has only three configurable parameters that allow the generation of parcellations with a smaller or larger number of sub-parcels.

Nevertheless, the limitations of diffusion MRI should always be considered when analyzing results based on dMRI tractography. This technique is used to non-invasively reconstruct the major white matter tracts of the brain. Tractography algorithms are able to generate valid bundles, however, due to the limited spatial resolution of the voxels and the large numbers of fiber pathways that can pass through them, false positives and false negatives are also generated. In fact, a non-negligible number of false positive bundles is produced, some of them reproducible across subjects. One of the next challenges of tractography will be to control these false positives and to improve the spatial reconstruction of existing WM tracts (Maier-Hein et al., 2017). Therefore, special care must be taken when interpreting the results given by tractography algorithms in a study. The differences in connectivity profiles can be produced due to artifacts in the tractography. The parcellations based on diffusion tractography are therefore only valid when the differences in connectivity profiles reflect true anatomical differences (Campbell and Pike, 2014). We have shown one example of biological significance, for the Broca's and Wernicke's areas, but further studies need to be performed to validate the diffusion-based parcellations.

### 4.1. Conclusions and Future Work

The proposed method creates a fine-grained parcellation of the cortical surface, consisting of the subdivision of coarse anatomical parcels, based on a diffusion-based fiber bundle atlas. The generated parcellation depends on configurable parameters that generate a parcellation with a smaller or larger number of sub-parcels. Furthermore, an intermediate output of the method is the probabilistic representation of the preliminary sub-parcels, associated to the two connections of each bundle. This information could be used, in combination with individual segmented bundles, to create individual parcellations, adapted to each subject. Its effect will be small changes on the boundaries of the sub-parcels of each subject, due to individual differences in the segmented bundles. Adapted parcellations should lead to increased consistency in structural connectome across subjects.

Moreover, other improvements could be implemented in future works. For example, a new atlas bundle could be used, based on a larger database, like the HCP database and probabilistic tractography. The bundles could also be obtained from an inter-subject fiber clustering from the same database, which could lead to a better representation of WM bundle connections of the population of subjects. However, the post-processing of candidate sub-parcels would probably be more complicated due to a larger amount of bundles and a higher sub-parcel overlapping.

Functional information could also be used to create a parcellation by using multimodal parcellation frameworks (Parisot et al., 2017). Furthermore, another line to explore is the inclusion of some atlas bundles based on known functional areas.

As a final conclusion, the proposed method can create a fine-grained cortical parcellation based on structural connectivity, from coarse anatomical parcels, leading to sub-parcels with high consistency in connectivity profiles across a population of subjects, and a degree of correspondence with state-of-the-art parcellations based on different modalities.

## Data Availability Statement

The data supporting the conclusions of this article will be made available by the authors, without undue reservation, to any qualified researcher.

## Ethics Statement

The studies involving human participants were reviewed and approved by Comité de Protection des Personnes Ile-de-France VII CPP100002/CPP100022, France. The patients/participants provided their written informed consent to participate in this study.

## Author Contributions

NL-L, AV, and PG designed the main research idea and wrote the manuscript. CP provided the pre-processed ARCHI database. J-FM, SL, and PG provided the guidance on the implementation and evaluation of the algorithms. JH provided the guidance on the overall work based on the main applications of the proposed method. NL-L and AV wrote the main analysis code and performed all the experiments. All authors read and approved the final manuscript.

## Conflict of Interest

The authors declare that the research was conducted in the absence of any commercial or financial relationships that could be construed as a potential conflict of interest.
