# Supplementary material for: From Coarse to Fine-Grained Parcellation of the Cortical Surface Using a Fiber-Bundle Atlas
Source: Front Neuroinform. 2020 Sep 10;14:32. doi: 10.3389/fninf.2020.00032 (PMC7533645; doi:10.3389/fninf.2020.00032)
Supplement: Supplementary file 1 [file Data_Sheet_1.pdf]

Supplementary Material

1 SUPPLEMENTARY FIGURES AND TABLES

1.1 Comparison of superficial white matter bundles for both SWM atlases

Figure S1 shows similar bundles between the two superficial white matter (SWM) atlases, *swm_atlas_1* (Guevara et al., 2017) and *swm_atlas_2* (Román et al., 2017). Three bundles of the left hemisphere (CMF-Op_0, CMF-PrC_0 and RMF-SF_0) and three bundles of the right hemisphere (IP-LO_0, PrC-SM_0, Tr-Ins_0) with high similarity, were taken as examples for comparison between both atlases.

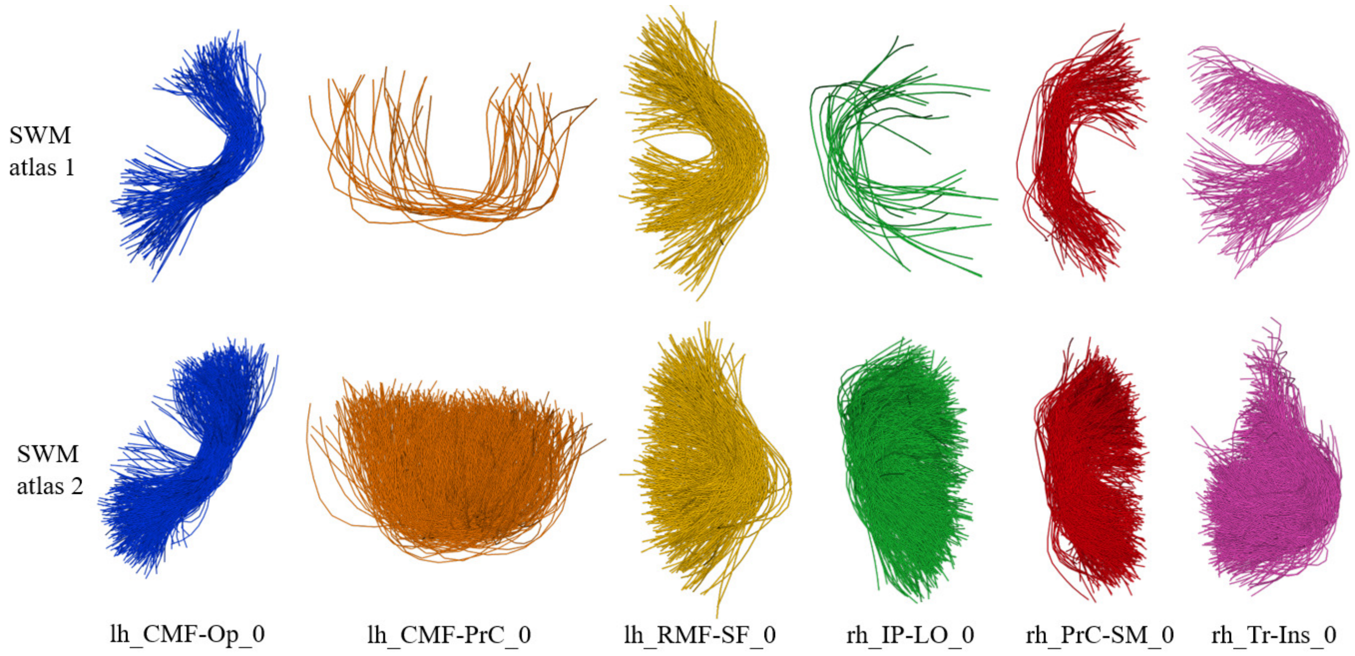

Figure S1. Some examples of bundles with high similarity between both SWM atlases. First row: *swm_atlas_1*, second row: *swm_atlas_2*.

Figure S2 displays the superficial white matter and deep white matter (DWM) bundles used to create the final fused white matter atlas.

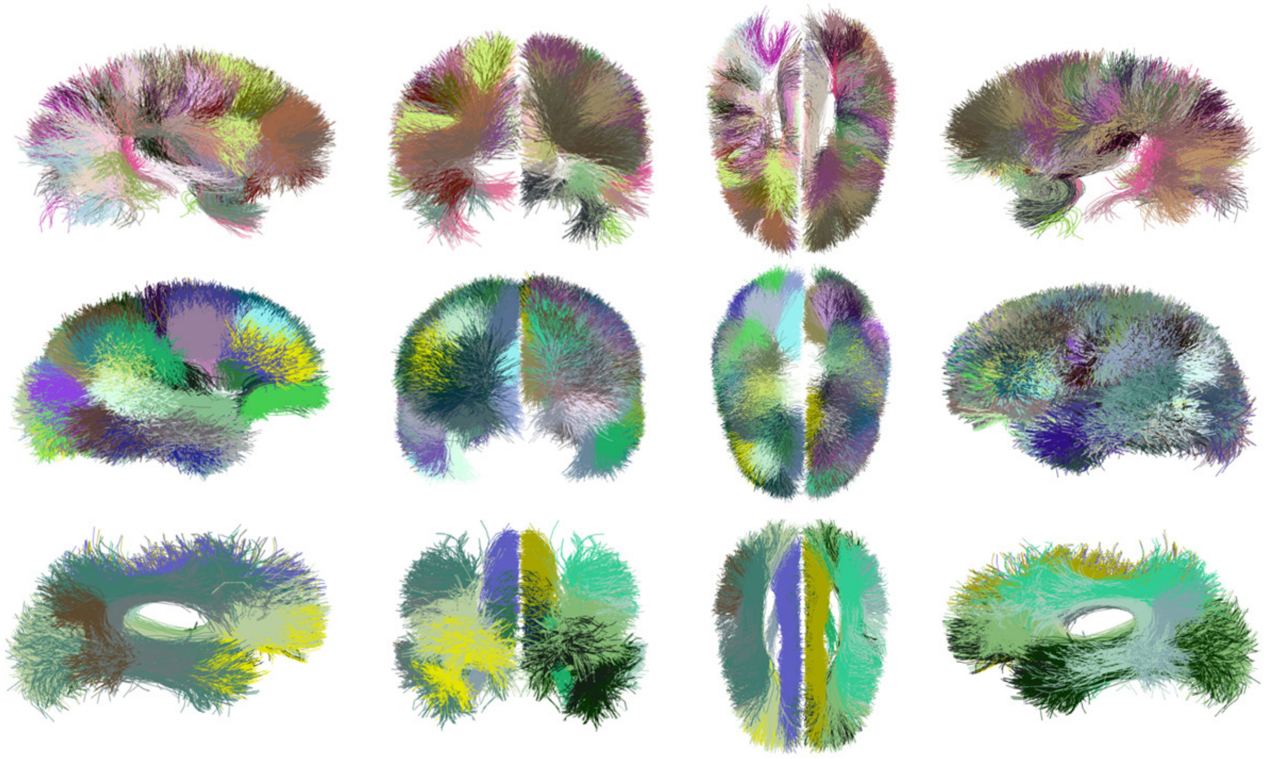

Figure S2. Bundles of the SWM and DWM atlases used for the creation of the fused final atlas. First row: *swm_atlas_1* (Guevara et al., 2017), composed of 50 bundles in each hemisphere. Second row: *swm_atlas_2* (Román et al., 2017), with 27 bundles in the left hemisphere and 34 in the right hemisphere, after selecting the bundles that complement *swm_atlas_1*. Third row: *DWM atlas* (Guevara et al., 2012), composed of 9 bundles per hemisphere. Right sagittal, coronal, axial and left sagittal views.

1.2 Fiber bundle segmentation

Figure S3 shows in the first row the final fused atlas of white matter bundles. The bottom row contains a segmented subject with the final atlas.

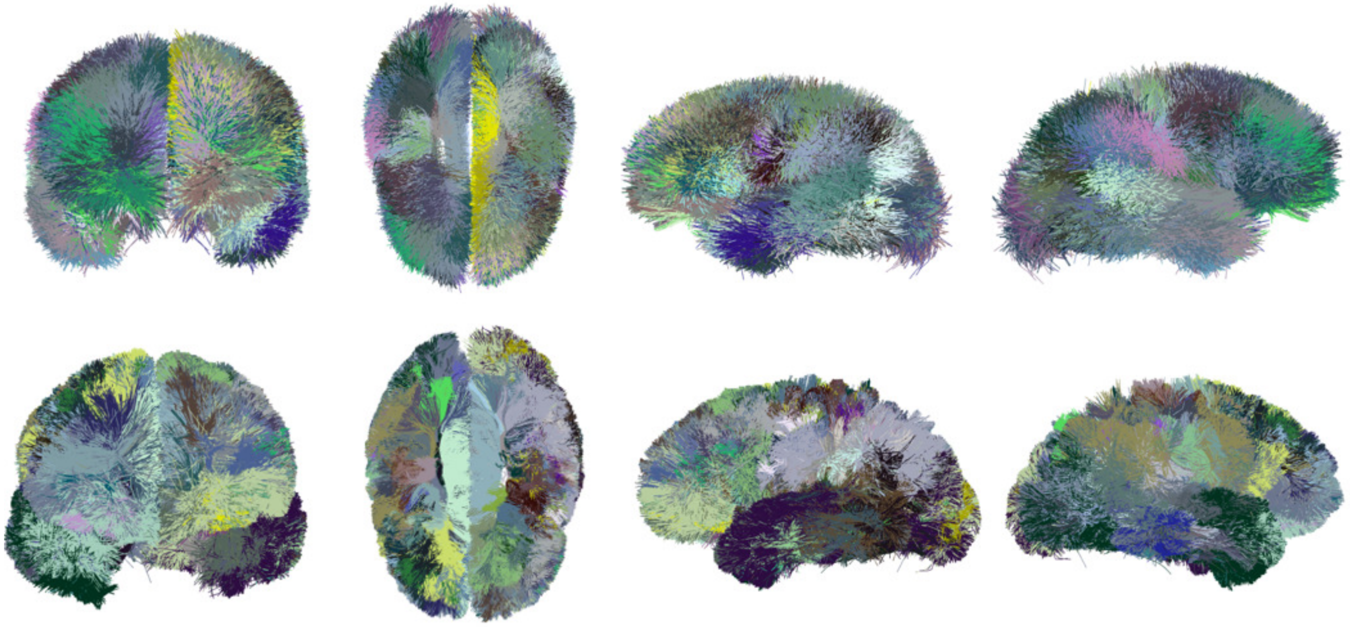

Figure S3. Example of bundle segmentation. The first row shows the final fused atlas of white matter bundles used by the proposed method. The second row shows a segmented subject, based on the atlas. Coronal, axial, left sagittal and right sagittal views.

1.3 Parameter configuration for parcellation creation

In this section, an example of the parameter $size\_thr$ is shown. Figure S4 illustrates the *Removing of small preliminary sub-parcels* sub-step, belonging to Step 5 of the parcellation method for the precentral anatomical parcel (PrC), by using $size\_thr$ = 0.10 and $size\_thr$ = 0.30.

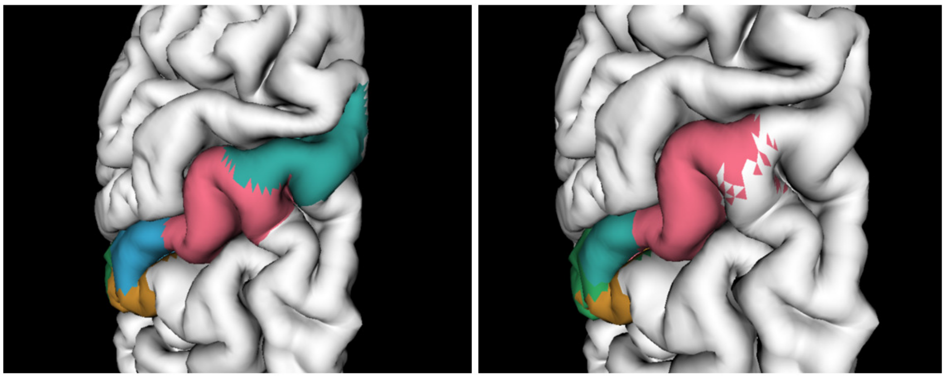

Figure S4. Example of size threshold for the removing of small preliminary sub-parcels ($size\_thr$), for the precentral (PrC) anatomical parcel. Left: $size\_thr$ = 0.1, Right: $size\_thr$ = 0.3. A small $size\_thr$ will prevent the removal of relatively big sub-parcels, which could leave uncovered regions in the cortex.

1.4 Parcel post-processing

This section shows the results of parcel post-processing (Step 6). Figure S5 gives an example of *Removing small connected components* sub-step, belonging to Step 6 of the proposed method (Sub-parcel post-processing). Finally, Figure S6 illustrates the final hard parcellation from *atlas 1* after applying *removing small connected components, erosion and dilation*.

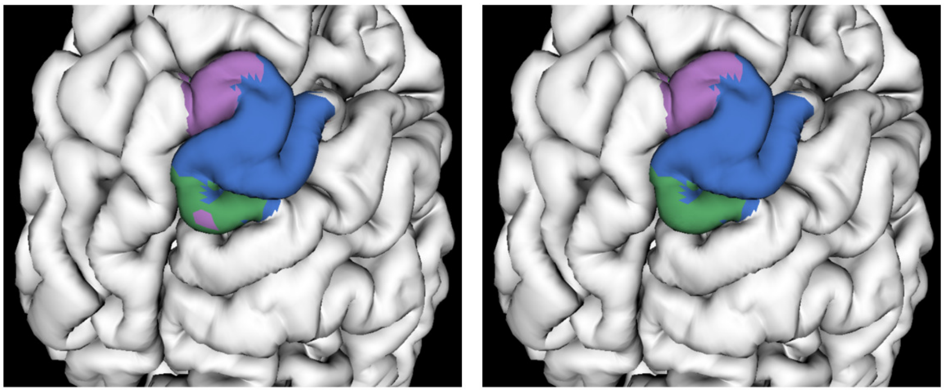

Figure S5. Removing small connected components (Step 6). The image on the left shows the SM anatomical parcel, divided into three sub-parcels with small connected components among them. Each connected component is represented with a color but internally is modeled as a graph. The image on the right shows the SM anatomical parcel after applying the removal of the small connected components, resulting in more uniform sub-parcels.

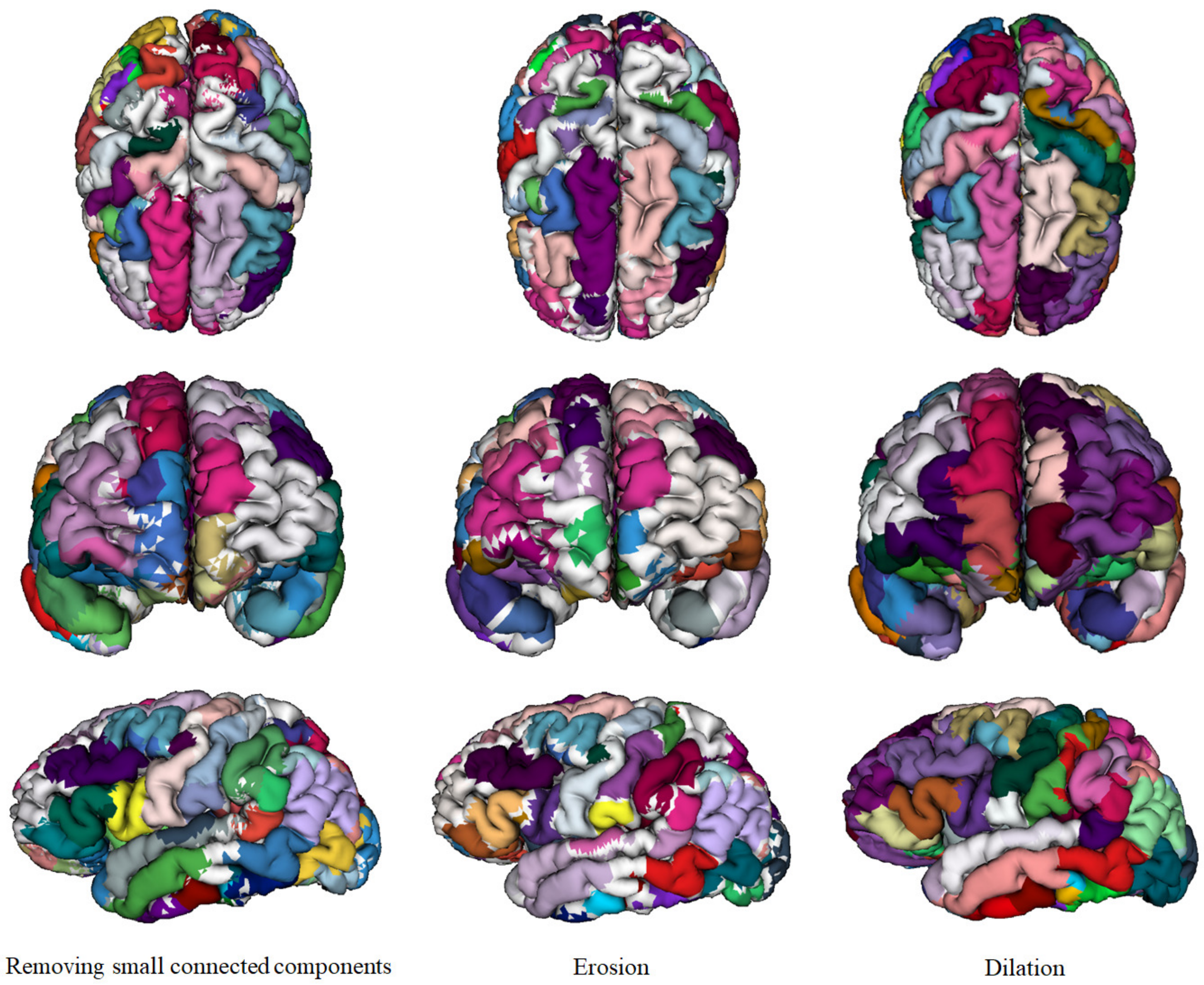

Figure S6. Results for Parcel post-processing (Step 6) stages. The first column shows the hard parcellation after applying the removal of the small connected components. The second column shows the result after applying erosion to the sub-parcels and the third column is the result of applying dilation. This is the final hard parcellation obtained, for $size\_thr = 0.1$, $dc\_thr = 0.1$ and $idc\_thr = 0.1$, consisting of 85 sub-parcels in the left hemisphere and 72 sub-parcels in the right hemisphere. For all the columns the axial, coronal and left sagittal views are displayed.

1.5 Brain graph construction

We performed some tests, based on graph network analysis. For that, the connectivity matrix of each of the 79 subjects was calculated for each cortical parcellation result. Each matrix was obtained using the generated parcellation, given by the sub-parcel labels, applied to the subject's cortical mesh, and the whole tractography of the subject. The constructed connectivity matrices are binary, denoting the existence or absence of a connection between the pair of sub-parcels, given by at least one fiber.

Complex networks in graph theory are modeled as a graph G formed by nodes $v \in V$ and linked by edges $e \in E$, such that $G = (V, E)$. Hence, each matrix was converted to a graph, for analyzing it using network graph metrics. Although using binary undirected graphs is a simplification of reality in terms of brain networks (Bassett et al., 2012), in neuroimaging it is an accepted technique because the signal-to-noise ratio is limited in the data (Achard et al., 2006).

Figure S7 shows the connectivity matrices for three subjects using *atlas 5*. Three matrices are displayed for each subject: binary, count (not binarized) and the logarithm count. Note that different values were tested to set the minimum number of fibers used to define the existence of a connection, leading to non-significant differences in the comparisons detailed below. As an example, Figure S8 shows the sub-parcels and connections obtained in *atlas 5*, for the precentral (PrC) and postcentral (PoC) gyri, associated with the main motor area and the primary somatosensory cortex, respectively (Catani, 2017).

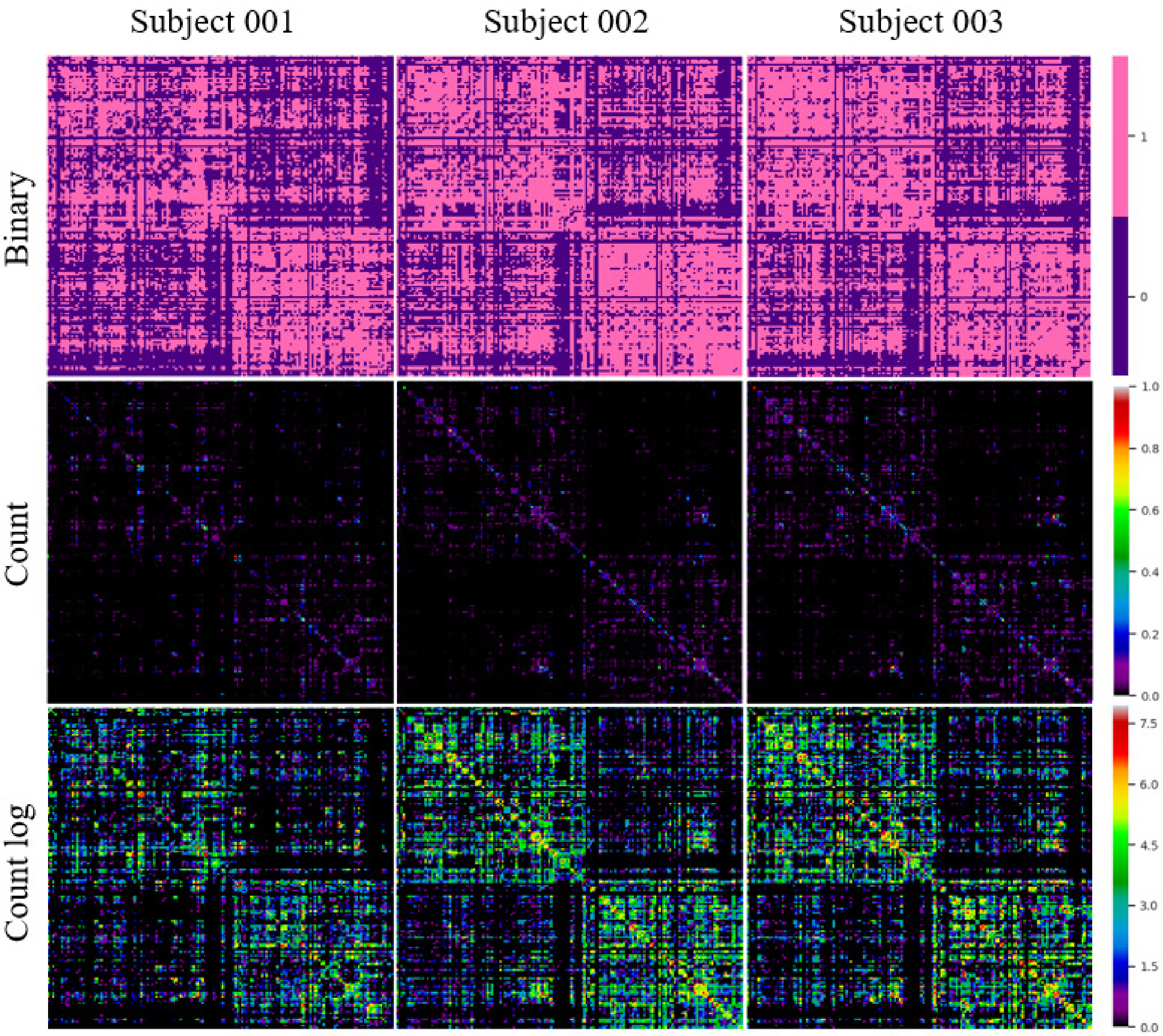

Figure S7. Connectivity matrices for three subjects, using *atlas 5*. The matrices are squared and have a dimension equal to the number of sub-parcels of the atlas. The first row represents the binary matrices, which indicate the presence or absence of a connection between the sub-parcels. The second row displays the count matrices, which contain the number of fibers connecting each pair of sub-parcels, normalized between 0 and 1. Finally, the third row displays the logarithmic count matrices, which contain the logarithm of the non-normalized count matrix. A high similarity can be seen between the different subjects.

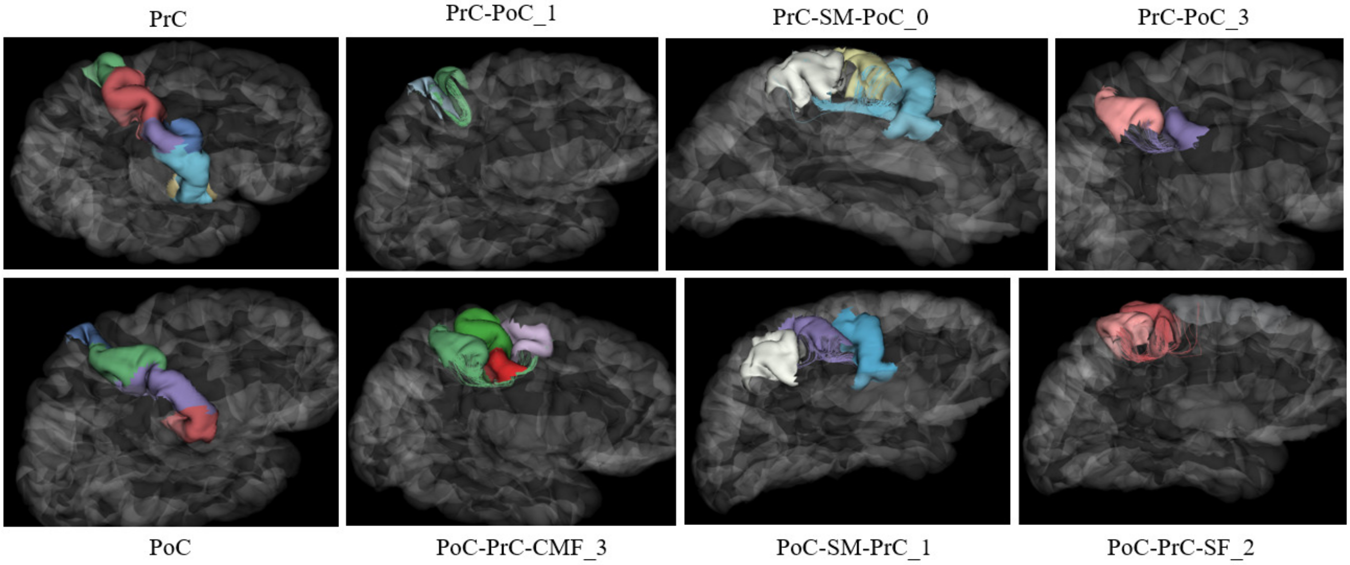

Figure S8. Example of structural connectivity between different cortical regions of the precentral (PrC) and postcentral (PoC) sub-parcels, according to *atlas 5*, based on parameter configuration given by Table 1 (from the main manuscript). Upper row: subdivision into sub-parcels of the precentral anatomical parcel, and the connections between these sub-parcels with other sub-parcels of PoC and SM (supramarginal) regions. Lower row: subdivision into sub-parcels of the postcentral anatomical parcel, and the connections between these sub-parcels with other sub-parcels of the CMF (caudal middle frontal), SM and SF (superior frontal) regions.

1.6 Brain network metrics

To conduct the study on the characteristics of a brain network, there is a wide range of metrics (Cohen and D'Esposito, 2016; Bullmore and Sporns, 2009). Three features, representatives of the parcellation problem were selected: functional segregation, functional integration, and small-worldness (Rubinov and Sporns, 2010). To perform the calculations, we used the *bctpy* library (https://github.com/aestrivex/bctpy), which is an adaptation of the Matlab Brain Connectivity Toolbox for Python (Rubinov and Sporns, 2010). Specifically, *bctpy* was used to calculate the clustering coefficient C (functional segregation) and path length L (functional integration) metrics. In addition, to evaluate the metric of small-world (ω coefficient), the *networkx* Python library (Telesford et al., 2011; Humphries et al., 2005; Humphries and Gurney, 2008) was employed. Supplementary material contains a description of each one of these metrics.

Clustering coefficient

This measure of functional segregation in the brain is defined as the existence of large highly connected groups of nodes or clusters in brain regions (Watts and Strogatz, 1998). It is a measure of the completeness of a node's neighborhood. The metric can be calculated for the entire network, as the average clustering coefficient. This metric ranges from 0 to 1. A low clustering coefficient defines a random network, while a complex network has a high clustering coefficient (Bullmore and Sporns, 2009). For undirected graphs, the clustering coefficient is defined by Equation S1:

$$C_i = \frac{2N_i}{k_i(k_i - 1)} \tag{S1}$$

where C_i is the clustering coefficient of a node i, N_i is the number of links between the neighbors of i, and k_i is the degree of node i. In graph theory, the degree of a node (or vertex) is the number of edges adjacent to the node, i. e. the number of nodes that are directly connected to the node. Equation S2 describes the average clustering coefficient that measures the whole network.

$$C = \frac{1}{n}\sum_{i \in G} C_i \tag{S2}$$

where C is the average clustering coefficient, G is the undirected network graph and n is the total number of nodes in the network.

Figure S9 (top left) shows a box plot for the average clustering coefficient C, obtained for each parcellation configuration (atlas), given by Table 1 (see main manuscript). Each box represents a generated parcellation. The median is displayed as a line inside the box, and the mean appears as a rhombus. The lines known as *mustaches* indicate the variability outside the lower quartile and the upper quartile. Finally, the red dots indicate the outliers in the data. Following the definition of the clustering coefficient, a complex network must have a high clustering coefficient, so in our chart, the generated parcellations that best meet the definition are *atlas 1* and *atlas 5*. Besides, they are the ones that show less variability. Note that a larger number of sub-parcels will give a lower value of C, which is congruent with the literature.

Path length

It is the most used measure for functional integration and measures the number of edges that exist to get from one node to another in the network. In other words, it is the easiness to distribute information among the different regions of the brain. The path length, also called *average shortest path length* or *characteristic*

path length (Watts and Strogatz, 1998), is calculated based on the shortest path length of each pair of nodes that make up the network. Lattice networks have long average path length, unlike complex networks or random networks that have short average path length (Bullmore and Sporns, 2009). For undirected graphs, the characteristic path length is defined by Equation S3:

$$L = \frac{1}{n(n-1)} \sum_{i,j \in G, i \neq j} d_{ij} \tag{S3}$$

where L is the characteristic path length, n is the total number of nodes in the network, G is the undirected network graph and d_{ij}, also known as *geodesic length*, is the shortest distance between nodes i and j.

Figure S9 (top right) shows the box plots obtained for the characteristic path length L, for each parcellation configuration (atlas). We can observe that the characteristic path length is proportionally lower than the respective average clustering coefficient, which complies with the characteristic that a complex network must have a small average shortest path length.

Small-worldness

All natural networks comply with the small-world topology showing their specific functionality by moving away from randomness. The small-worldness metric combines a high clustering coefficient (functional segregation) while keeping a low characteristic path length (functional integration), which links all the nodes in the network (Bullmore and Sporns, 2009). In other words, all nodes are locally strongly interconnected, and they are linked to other regions through a few links.

Small-worldness can be quantified with the coefficient called ω (Telesford et al., 2011). It is calculated comparing the clustering coefficient of the analyzed network (C) to an equivalent lattice network (C_l), and its path length (L) to an equivalent random network (L_r), as described in Equation S4.

$$\omega = \frac{L_r}{L} - \frac{C}{C_l} \tag{S4}$$

When using the clustering coefficient of a lattice network, this metric is less sensitive to the fluctuations than the clustering coefficient of a random network. The ω coefficient ranges from -1 to 1 regardless of the size of the network. The closer the value of ω coefficient is to zero, the network is considered closer to small-world property, that is, $L \approx L_r$ and $C \approx C_l$ (Telesford et al., 2011).

Figure S9 (bottom) shows the ω coefficient for the different parcellation configurations (atlas). This coefficient has to be as close as possible to zero, to present the property of a small-world network. All parcellations comply with this property.

Best parcellation configuration according to brain network metrics

Taking into account the previous results, the configuration of parameters that achieve the best results, according to the small-world ω coefficient, are *atlas 1* and *atlas 5*. As shown, *atlas 1* and *atlas 5* have the value of ω coefficient closest to zero, presenting the best small-world network property. This is mainly due to the parameters dc_thr (density center threshold) and idc_thr (intersection of density centers threshold) since as we increase these two thresholds, fewer intersections are considered significant and fewer sub-parcels are merged. As known, fewer sub-parcels will lead to smaller path lengths, better complying with functional integration and a small shortest path length. Given that the size of the network (number of sub-parcels) is extremely determinant in small-world values, the previous results only allow us to conclude

that all networks tested have properties characteristic to small-world networks, being better for networks with fewer nodes. However, obtaining a large number of sub-parcels could be a desired feature, for example, for the analysis of small functional areas.

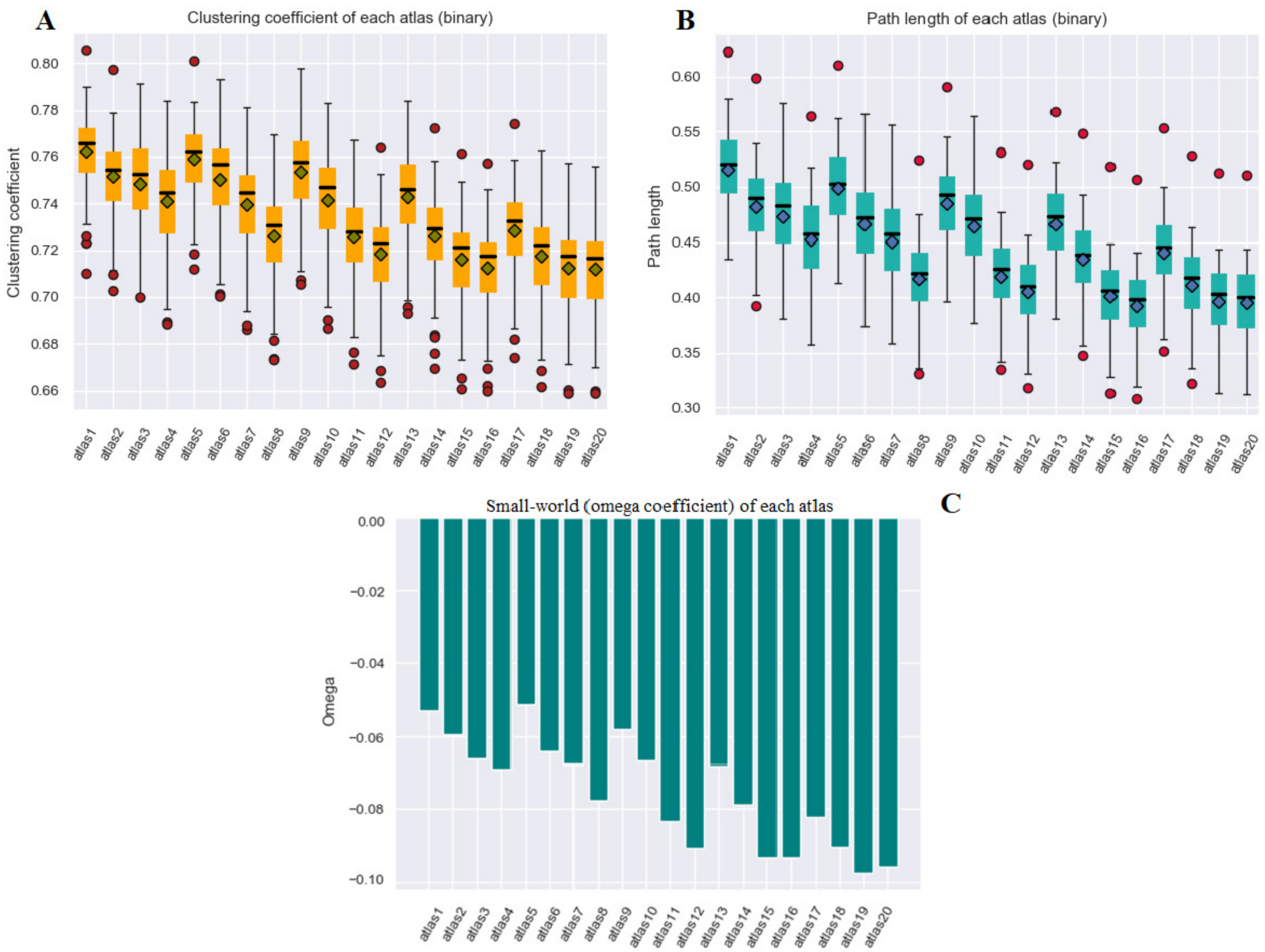

Figure S9. Metrics of brain networks. **(A)** Average clustering coefficient C for all parcellation (atlas) configurations, given by Table 1 (from main manuscript). *Atlas 1* and *atlas 5* achieve the best results for the clustering coefficient, being also those that have less variability. **(B)** Path length L for all parcellation (atlas) configurations, given by Table 1. For all atlases, the average shortest path length is small, complying with the complex network definition. **(C)** Small-world ω coefficient for all parcellation (atlas) configurations, given by Table 1. *Atlas 5* appears to be slightly better, since the closer to zero, the more it complies with the property of small-world.

1.7 Cross-validation analysis

To test the dependence of the proposed method on the input data, we applied the method to the ARCHI database using k-fold cross-validation (Kohavi et al., 1995). We created eight different datasets, composed of 69 subjects for the parcellation creation (atlas datasets), and the 10 remaining subjects for testing (testing datasets). The parameters used are those for *atlas 5*, i. e. $dc\_thr = 0.15$ and $idc\_thr = 0.10$. For more details, Table S7 lists the subjects used for each dataset.

The cortical parcellation method was applied to the eight atlas datasets. Next, each created parcellation was applied to the 10 subjects of the corresponding testing dataset and the connectivity matrix of each subject was calculated according to the corresponding parcellation. Finally, the different graph network metrics were calculated for each subject (average Dice coefficient, average clustering coefficient, path length, small-world ω coefficient), and averaged for each testing dataset. In general, a high similarity between the metrics for the different parcellations was found. The average Dice coefficient presented an average between 0.70 and 0.76, which indicates a good similarity of the connectivity matrix between subjects for all the parcellations, even though some testing datasets lead to better results.

To evaluate the influence of the testing dataset on the average Dice coefficient, we calculated the connectivity matrix and Dice coefficient for the whole dataset (79 subjects), separately for each one of the eight atlases generated. We found a higher similarity between subjects, with an average Dice coefficient varying between 0.72 and 0.74. Finally, the other metrics also showed high similarity, with an average clustering coefficient between 0.76 and 0.77, an average path length between 0.50 and 0.54, and small-world ω coefficient between -0.04 and -0.08. Hence, all networks comply with the small-world property.

Figures S10 and S11 show the metrics of Dice similarity, clustering coefficient, path length and small-world ω coefficient applied to the testing datasets (Figure S10) and the whole dataset (S11) for the eight atlases created, according to Table S7.

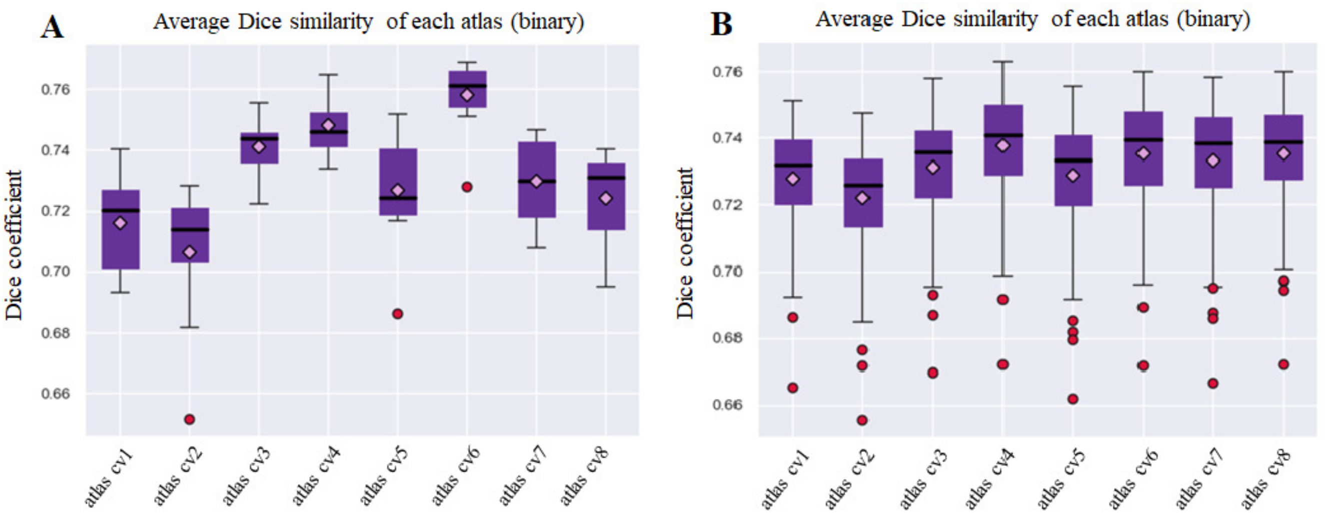

Figure S10. **(A)** Cross-validation analysis: Dice coefficient for the 10 subjects of the testing datasets, for each generated atlas, according to Table S7. Results show high similarity between subjects for all the atlases, even though some variability is found between the testing datasets. **(B)** Cross-validation analysis: Dice coefficient for all subjects using each generated atlas, according to Table S7. A lower variability between subjects was found between the different atlases when using the 79 subjects.

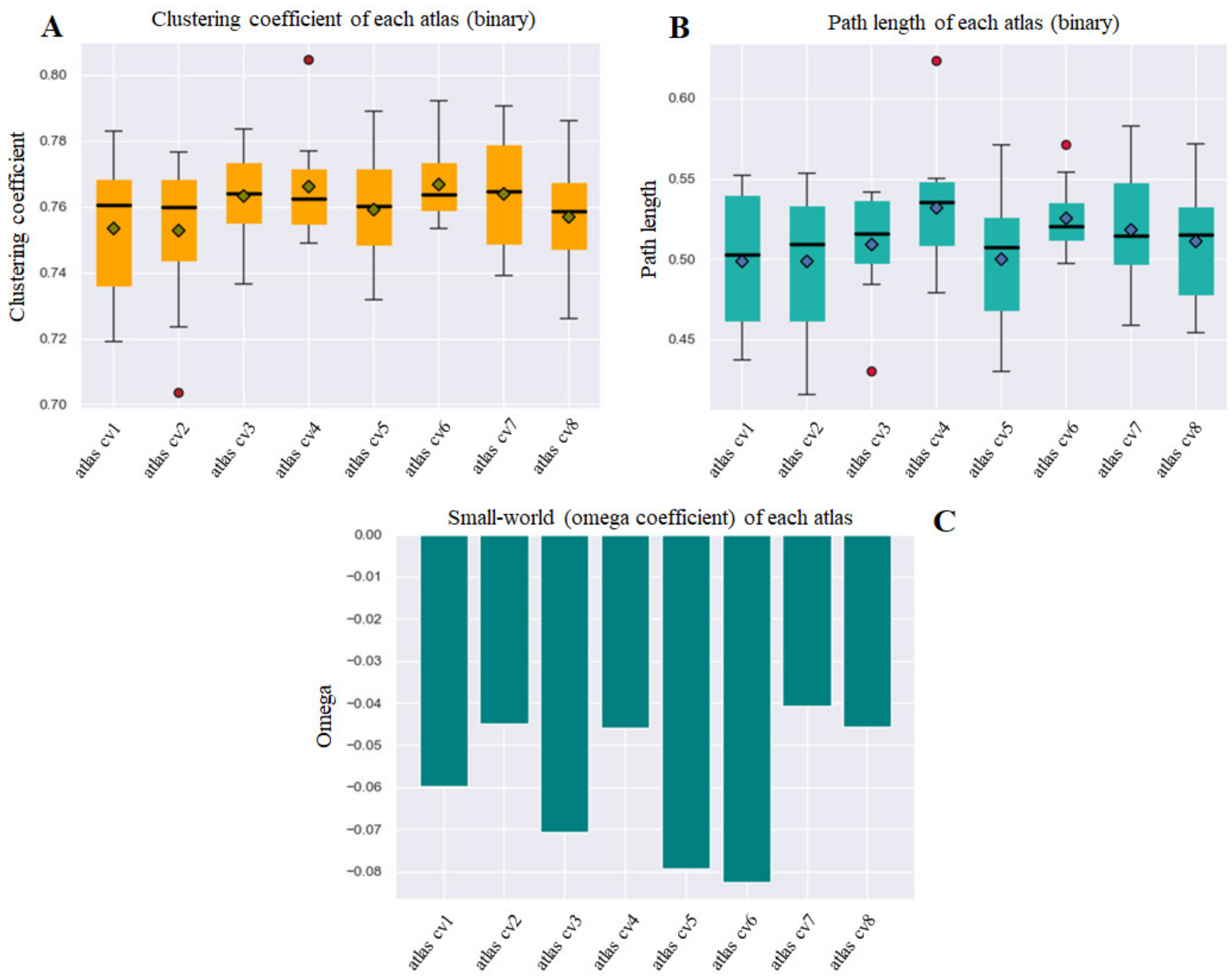

Figure S11. Cross-validation analysis: network metrics obtained for the 10 subjects of the testing datasets, for each generated atlas, according to Table S7. **(A)** Clustering coefficient. **(B)** Path length. **(C)** Small-world ω coefficient. In all the cases, results show a low variability between the different testing datasets. Also, an ω coefficient very close to zero was found for all the datasets, complying with the small-world property.

1.8 From Desikan-Killiany atlas to a finer granularity, example for *atlas 5*

Figure S12 illustrates the result of the parcellation obtained for *atlas 5* as well as *Desikan-Killiany* atlas. The first two rows contain the *Desikan-Killiany* parcellation, composed of 35 parcels per hemisphere. Rows three and four show the resulting parcellation for *atlas 5*, that subdivides the *Desikan-Killiany* atlas, based on white matter fiber connectivity. The parcellation contains 160 sub-parcels, 86 sub-parcels in the left hemisphere and 74 sub-parcels in the right hemisphere. For more details, see Table S4, which illustrates the number of sub-parcels obtained for each anatomical region in *atlas 5*, for each hemisphere.

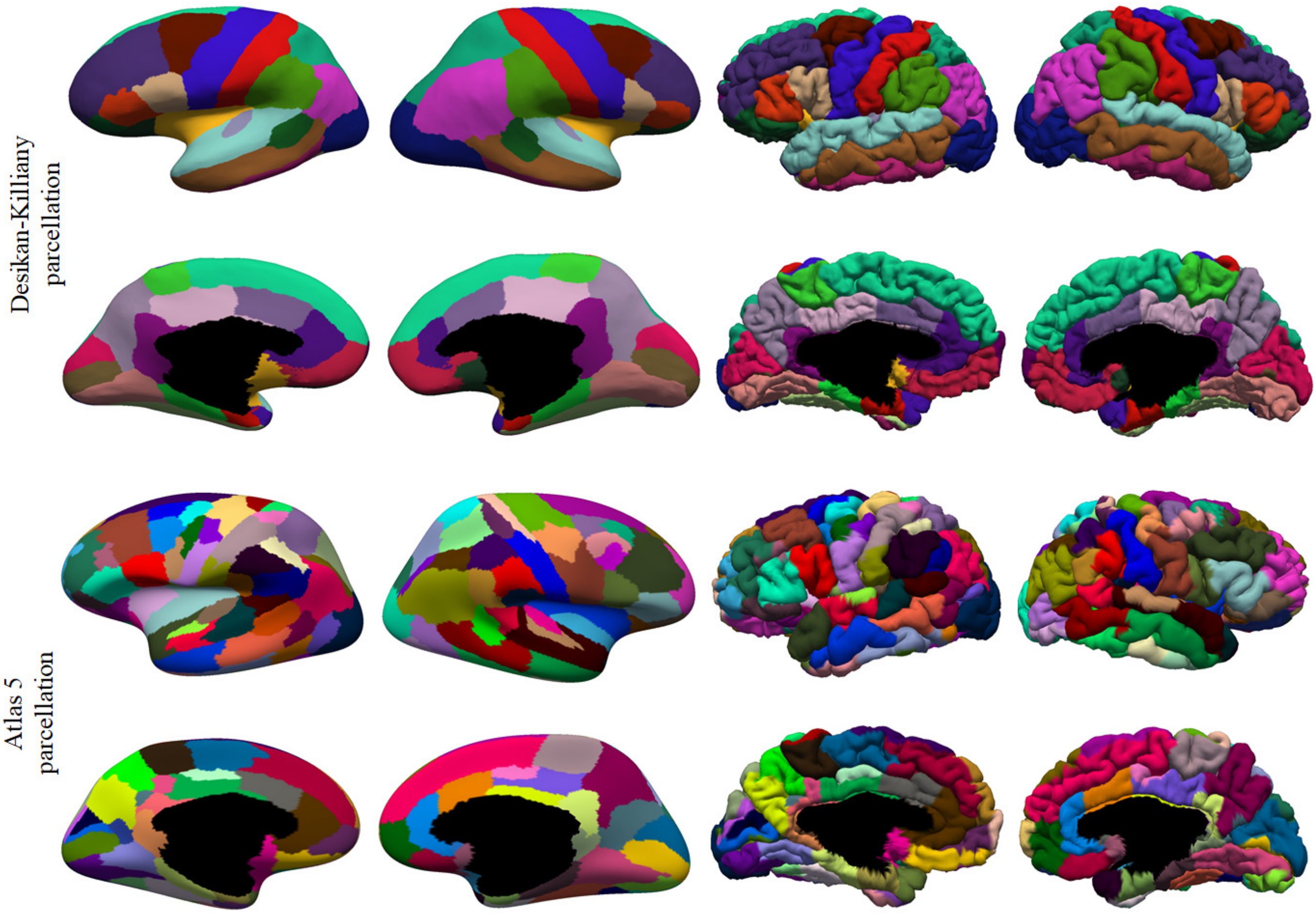

Figure S12. *Desikan-Killiany* atlas and *atlas 5*, obtained using the parameter configuration given by Table 1. The first and second rows show the *Desikan-Killiany* atlas, which is formed by 70 parcels, 35 in each hemisphere. Rows three and four illustrate *atlas 5* parcellation which is composed of 160 sub-parcels, 86 sub-parcels in the left hemisphere and 74 sub-parcels in the right hemisphere. Columns one and two show both parcellations with the inflated surface, while columns three and four show the pial surface.

1.9 Comparisons with state-of-the-art parcellations based on MRI modalities

Figure S13 shows the comparisons performed between *atlas 5* and *Schaefer* (100 parcels), *PrAGMATiC* (320 parcels) and *Yeo* (7 or 17 networks) parcellations. Moreover, Figure S14 illustrates the comparisons between *atlas 13* and *Glasser* (360 parcels), *PrAGMATiC* and *Schaefer* (200 parcels) parcellations. Only parcels with a Dice's coefficient ≥ 0.6 are shown.

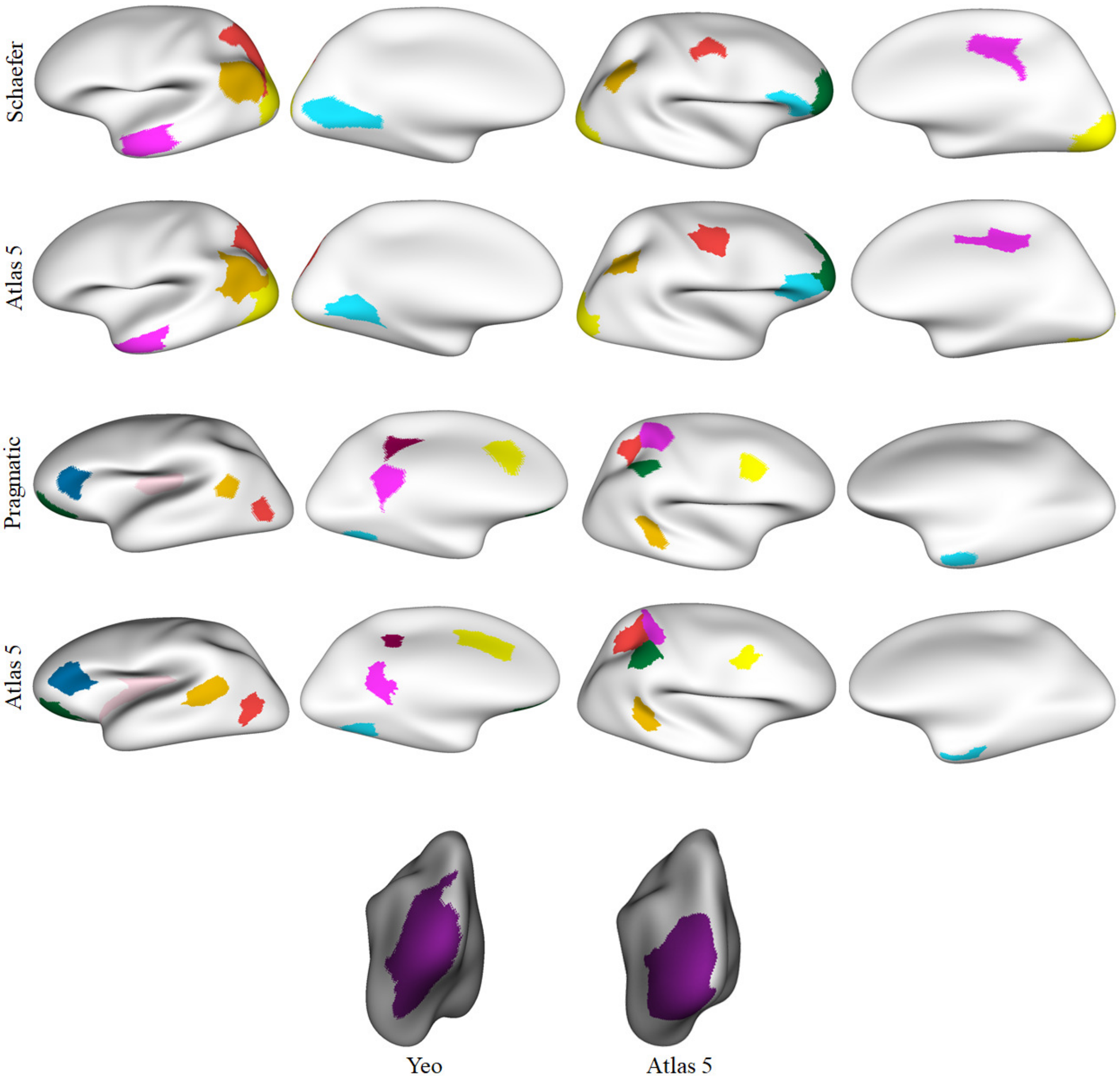

Figure S13. Parcels in common between *atlas 5* and state-of-the-art parcellations based on MRI modalities, with a Dice coefficient ≥ 0.6. Comparisons are shown for *Schaefer* (100 parcels), *PrAGMATiC* and *Yeo* atlases. All the meshes are inflated. *Schaefer* has 11 parcels in common with *atlas 5*, 5 in the left hemisphere and 6 in the right hemisphere. *PrAGMATiC* has 15 similar parcels with *atlas 5*, 9 in the left hemisphere and 6 in the right hemisphere. Finally, *Yeo* atlas with 17 networks has one parcel in common with *atlas 5* in the left hemisphere.

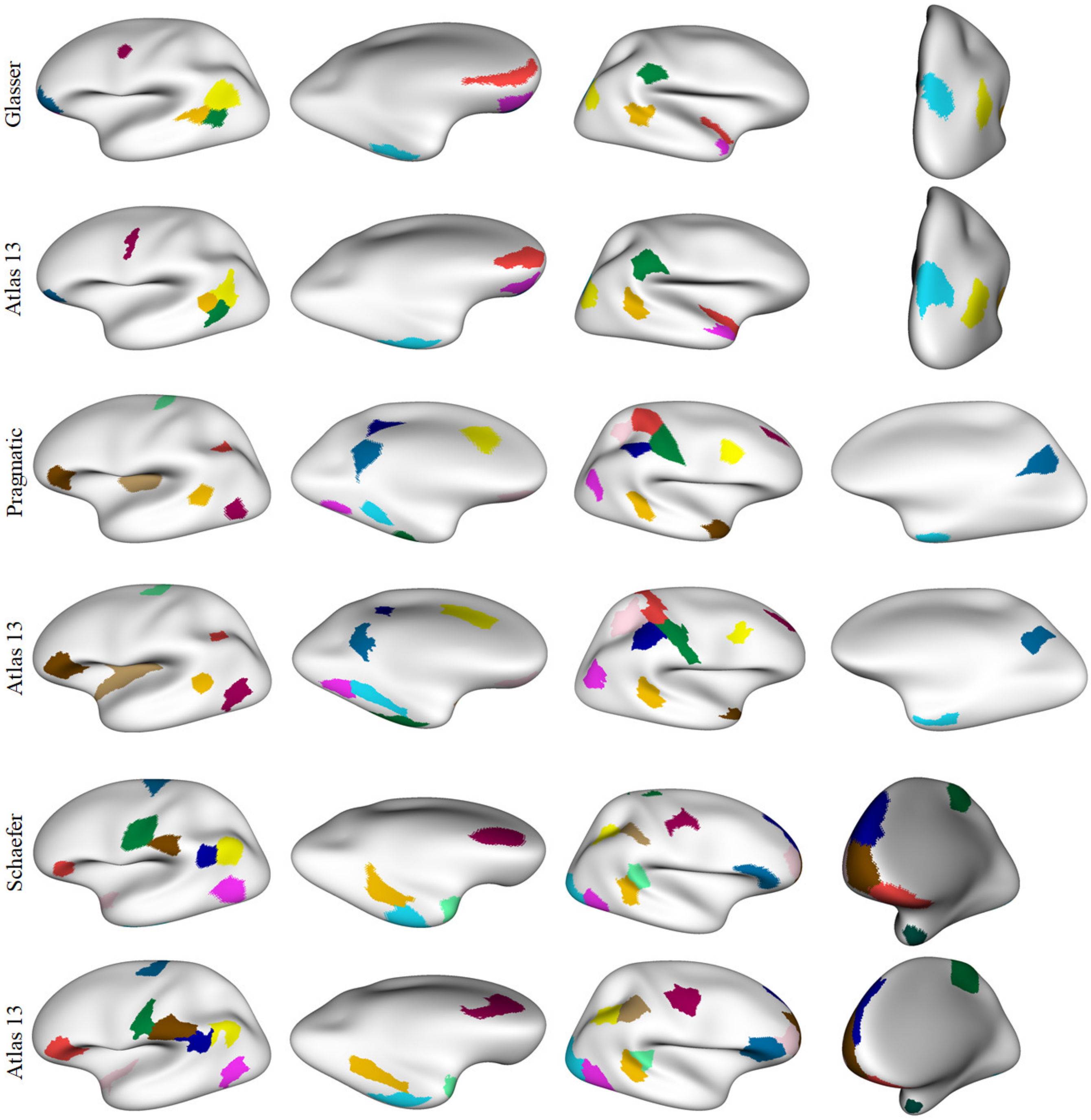

Figure S14. Common parcels between *atlas 13* and state-of-the-art parcellations based on MRI modalities, with a Dice coefficient ≥ 0.6. Comparisons are shown for *Glasser*, *PrAGMATiC* and *Schaefer* atlases. All the meshes are inflated. *Glasser* has 14 parcels in common with *atlas 13*, 8 in the left hemisphere and 6 in the right hemisphere. On the other hand, *PrAGMATiC* has 22 similar parcels with *atlas 13*, 12 parcels in the left hemisphere and 10 parcels in the right hemisphere. Finally, *Schaefer* parcellation (200 parcels) has 26 common parcels, 12 the left hemisphere and 14 in the right hemisphere.

1.10 Complementary Tables

Tables S1 and S2 list the bundles of the final fused atlas for the left and right hemispheres, respectively. The left hemisphere is composed of 50 bundles of *swm_atlas_1*, 27 bundles of *swm_atlas_2* and nine of DWM atlas. On the other hand, the right hemisphere contains 50 bundles of *swm_atlas_1*, 34 bundles of *swm_atlas_2* and nine of DWM atlas.

Table S3 contains the 35 regions (gyri) and abbreviations of the *Desikan-Killiany* atlas (Desikan et al., 2006).

Table S4 lists for each hemisphere the number of sub-parcels obtained for *atlas 5* and *atlas 13*, respectively, for each anatomical region (gyrus) after applying the parcellation method. On the other hand, Table S5 shows the sub-parcels that *atlas 13* has in common with the other atlases generated by the method, for a Dice coefficient ≥ 0.6.

Table S6 illustrates the comparison between *atlas 5*, *atlas 13*, *Lefranc* and *Brainnetome* on the number of parcels per anatomical parcel, based on *Desikan-Killiany (DK)* atlas. All the atlases use *DK* atlas as a coarse anatomical parcellation, but *Braintomme* is based on some regions of this atlas that are combined. Therefore in some cases, *Braintomme* parcels cannot be perfectly matched as subdivisions of *DK* standard regions. The * is used to indicate the *DK* anatomical parcels where Brainnetome performs a different subdivision of the regions and only an approximate number of subdivisions is provided. In the following, we briefly describe these cases.

The Pars Opercularis and the Pars Triangularis *DK* atlas regions correspond to the Inferior Frontal Gyrus (IFG) in *Brainnetome*, that is divided into six parcels. Three parcels correspond approximately to the Pars Opercularis and the other three parcels to the Pars Triangularis.

The Orbital Gyrus (OrG) in *Brainnetome* is subdivided into six parcels, that correspond to the Pars Orbitalis (one parcel), Frontal Pole (one parcel), Lateral Orbito Frontal (one parcel), and Medial Orbito Frontal(three parcels).

The Superior Temporal, Transverse Temporal, and Temporal Pole *DK* anatomical parcels correspond to the Superior Temporal Gyrus (STG) in *Brainnetome*. This region is subdivided into six parcels, having a good correspondence with the Superior Temporal (four parcels), the Temporal Pole (one parcel), and the Transverse Temporal (one parcel) regions.

The Parahippocampal Gyrus (PhG) in the *Brainnetome* atlas is subdivided into six parcels, corresponding to the Entorhinal (two parcels) and Parahippocampal (four parcels) *DK* anatomical regions.

The *DK* Inferior Parietal and Supramarginal anatomical parcels correspond to the Inferior Parietal Lobule (IPL) in *Brainnetome*, where each *DK* region is subdivided into three parcels.

The MedioVentral Occipital Cortex (MVOcC) of *Brainnetome* atlas is subdivided into five parcels. These correspond to two parcels for the Cuneus, one parcel for the Pericalcarine, and one parcel for the Lingual region.

Finally, the Cingulate Gyrus (CG) in *Brainnetome* is composed of 7 parcels. These parcels correspond in *DK* atlas to two parcels for the Posterior Cingulate, two parcels for the Isthmus, two parcels for the Anterior Cingulate and one parcel for the Rostral Anterior Cingulate region.

See the supplementary material[1] of "The Human Brainnetome Atlas" for more information on equivalences between atlases.

Finally, Table S7 lists the datasets used for cross-validation analysis from the 79 subjects of the ARCHI database.

Table S1. Bundles of the left hemisphere of the final fused bundle atlas. The name of the bundles in the original SWM and DWM atlases and the name of the atlas to which the bundles belong are listed. Nine bundles are from DWM atlas, 50 bundles are from *swm_atlas_1* and 27 bundles are from *swm_atlas_2*.

Bundle	Atlas	Bundle	Atlas	Bundle	Atlas
AR_ANT_LEFT	DWM	lh_IT-IT_1l	*swm_atlas_2*	lh_PoC-SM_0	*swm_atlas_1*
AR_LEFT	DWM	lh_IT-MT_0	*swm_atlas_1*	lh_PoC-SM_1	*swm_atlas_1*
AR_POST_LEFT	DWM	lh_LOF-LOF_0i	*swm_atlas_2*	lh_PrC-Ins_0	*swm_atlas_1*
CG_LEFT	DWM	lh_LOF-Or_0	*swm_atlas_1*	lh_PrC-PrC_0l	*swm_atlas_2*
CG2_LEFT	DWM	lh_LOF-RMF_0	*swm_atlas_1*	lh_PrC-SF_0	*swm_atlas_1*
CG3_LEFT	DWM	lh_LOF-RMF_1	*swm_atlas_1*	lh_PrC-SM_0	*swm_atlas_1*
IFO_LEFT	DWM	lh_LOF-ST_0	*swm_atlas_1*	lh_PrC-SM_1i	*swm_atlas_2*
IL_LEFT	DWM	lh_LO-LO_0i	*swm_atlas_2*	lh_PrCu-PrCu_0l	*swm_atlas_2*
lh_CAC-PrCu_0	*swm_atlas_1*	lh_LO-LO_1i	*swm_atlas_2*	lh_RAC-SF_1	*swm_atlas_1*
lh_CMF-CMF_0i	*swm_atlas_2*	lh_LO-LO_2l	*swm_atlas_2*	lh_RMF-RMF_0i	*swm_atlas_2*
lh_CMF-Op_0	*swm_atlas_1*	lh_MOF-ST_0	*swm_atlas_1*	lh_RMF-RMF_1i	*swm_atlas_2*
lh_CMF-PoC_0	*swm_atlas_1*	lh_MT-MT_0i	*swm_atlas_2*	lh_RMF-SF_0	*swm_atlas_1*
lh_CMF-PrC_0	*swm_atlas_1*	lh_MT-MT_1i	*swm_atlas_2*	lh_RMF-SF_1	*swm_atlas_1*
lh_CMF-PrC_1	*swm_atlas_1*	lh_MT-MT_1l	*swm_atlas_2*	lh_SF-SF_0l	*swm_atlas_2*
lh_CMF-RMF_0	*swm_atlas_1*	lh_MT-SM_0	*swm_atlas_1*	lh_SM-Ins_0	*swm_atlas_1*
lh_CMF-SF_0	*swm_atlas_1*	lh_MT-ST_0	*swm_atlas_1*	lh_SM-SM_0i	*swm_atlas_2*
lh_Cu-Li_0l	*swm_atlas_2*	lh_Op-Ins_0	*swm_atlas_1*	lh_SM-SM_1i	*swm_atlas_2*
lh_Fu-Fu_0i	*swm_atlas_2*	lh_Op-PrC_0	*swm_atlas_1*	lh_SM-SM_2i	*swm_atlas_2*
lh_Fu-Fu_1l	*swm_atlas_2*	lh_Op-SF_0	*swm_atlas_1*	lh_SP-SM_0	*swm_atlas_1*
lh_Fu-IT_0i	*swm_atlas_2*	lh_Or-Ins_0	*swm_atlas_1*	lh_SP-SP_0i	*swm_atlas_2*
lh_Fu-LO_0	*swm_atlas_1*	lh_PoC-Ins_0	*swm_atlas_1*	lh_ST-Ins_0	*swm_atlas_1*
lh_IC-PrCu_0	*swm_atlas_1*	lh_PoCi-PrCu_0	*swm_atlas_1*	lh_ST-ST_0i	*swm_atlas_2*
lh_IP-IT_0	*swm_atlas_1*	lh_PoCi-PrCu_1	*swm_atlas_1*	lh_ST-ST_1l	*swm_atlas_2*
lh_IP-LO_1	*swm_atlas_1*	lh_PoCi-RAC_0	*swm_atlas_1*	lh_ST-TT_0	*swm_atlas_1*
lh_IP-MT_0	*swm_atlas_1*	lh_PoCi-SF_0	*swm_atlas_1*	lh_Tr-Ins_0	*swm_atlas_1*
lh_IP-SM_0	*swm_atlas_1*	lh_PoC-PrC_0	*swm_atlas_1*	lh_Tr-RMF_0i	*swm_atlas_2*
lh_IP-SP_0	*swm_atlas_1*	lh_PoC-PrC_1	*swm_atlas_1*	lh_Tr-SF_0	*swm_atlas_1*
lh_IP-SP_1	*swm_atlas_1*	lh_PoC-PrC_2	*swm_atlas_1*	UN_LEFT	DWM
lh_IT-IT_0l	*swm_atlas_2*	lh_PoC-PrC_3	*swm_atlas_1*		

[1] https://www.ncbi.nlm.nih.gov/pmc/articles/PMC4961028/

Table S2. Bundles of the right hemisphere of the final fused bundle atlas. The name of the bundles in the original SWM and DWM atlases and the name of the atlas to which the bundles belong are listed. Nine bundles are from DWM atlas, 50 bundles are from *swm_atlas_1* and 34 bundles are from *swm_atlas_2*.

Bundle	Atlas	Bundle	Atlas	Bundle	Atlas
AR_ANT_RIGHT	DWM	rh_LOF-LOF_1r	*swm_atlas_2*	rh_PrC-Ins_0	*swm_atlas_1*
AR_POST_RIGHT	DWM	rh_LOF-MOF_0	*swm_atlas_1*	rh_PrC-SF_0i	*swm_atlas_2*
AR_RIGHT	DWM	rh_LOF-RMF_0	*swm_atlas_1*	rh_PrC-SM_0	*swm_atlas_1*
CG_RIGHT	DWM	rh_LOF-RMF_1	*swm_atlas_1*	rh_PrC-SM_1i	*swm_atlas_2*
CG2_RIGHT	DWM	rh_LOF-ST_0	*swm_atlas_1*	rh_PrC-SP_0	*swm_atlas_1*
CG3_RIGHT	DWM	rh_LO-LO_0i	*swm_atlas_2*	rh_PrCu-PrCu_0r	*swm_atlas_2*
IFO_RIGHT	DWM	rh_LO-LO_1i	*swm_atlas_2*	rh_RAC-SF_0	*swm_atlas_1*
IL_RIGHT	DWM	rh_LO-SP_0	*swm_atlas_1*	rh_RMF-RMF_0i	*swm_atlas_2*
rh_CAC-PoCi_0	*swm_atlas_1*	rh_MOF-ST_0	*swm_atlas_1*	rh_RMF-RMF_0r	*swm_atlas_2*
rh_CAC-PrCu_0	*swm_atlas_1*	rh_MT-MT_0i	*swm_atlas_2*	rh_RMF-RMF_1i	*swm_atlas_2*
rh_CMF-CMF_0i	*swm_atlas_2*	rh_MT-MT_0r	*swm_atlas_2*	rh_RMF-RMF_1r	*swm_atlas_2*
rh_CMF-Op_0i	*swm_atlas_2*	rh_MT-MT_1i	*swm_atlas_2*	rh_RMF-SF_0	*swm_atlas_1*
rh_CMF-PrC_0	*swm_atlas_1*	rh_MT-SM_0	*swm_atlas_1*	rh_RMF-SF_0r	*swm_atlas_2*
rh_CMF-PrC_1	*swm_atlas_1*	rh_MT-ST_0	*swm_atlas_1*	rh_RMF-SF_1	*swm_atlas_1*
rh_CMF-RMF_0	*swm_atlas_1*	rh_Op-Ins_0	*swm_atlas_1*	rh_SF-SF_1r	*swm_atlas_2*
rh_CMF-SF_0	*swm_atlas_1*	rh_Op-PrC_0	*swm_atlas_1*	rh_SF-SF_2r	*swm_atlas_2*
rh_CMF-SF_1	*swm_atlas_1*	rh_Op-SF_0	*swm_atlas_1*	rh_SM-Ins_0	*swm_atlas_1*
rh_Cu-Li_0	*swm_atlas_1*	rh_Op-Tr_0	*swm_atlas_1*	rh_SM-SM_0i	*swm_atlas_2*
rh_Fu-Fu_0i	*swm_atlas_2*	rh_Or-Ins_0	*swm_atlas_1*	rh_SM-SM_1i	*swm_atlas_2*
rh_Fu-IT_0i	*swm_atlas_2*	rh_PoCi-PrCu_1	*swm_atlas_1*	rh_SM-SM_2i	*swm_atlas_2*
rh_Fu-LO_1	*swm_atlas_1*	rh_PoCi-PrCu_2	*swm_atlas_1*	rh_SP-SM_0	*swm_atlas_1*
rh_IC-PrCu_0	*swm_atlas_1*	rh_PoCi-RAC_0	*swm_atlas_1*	rh_SP-SP_0i	*swm_atlas_2*
rh_IP-IP_0r	*swm_atlas_2*	rh_PoC-PoC_1r	*swm_atlas_2*	rh_SP-SP_0r	*swm_atlas_2*
rh_IP-IT_0	*swm_atlas_1*	rh_PoC-PrC_0	*swm_atlas_1*	rh_ST-ST_0i	*swm_atlas_2*
rh_IP-LO_0	*swm_atlas_1*	rh_PoC-PrC_1	*swm_atlas_1*	rh_ST-TT_0	*swm_atlas_1*
rh_IP-MT_0	*swm_atlas_1*	rh_PoC-PrC_1r	*swm_atlas_2*	rh_Tr-Ins_0	*swm_atlas_1*
rh_IP-SM_0	*swm_atlas_1*	rh_PoC-PrC_2	*swm_atlas_1*	rh_Tr-RMF_0i	*swm_atlas_2*
rh_IP-SP_0	*swm_atlas_1*	rh_PoC-PrC_3i	*swm_atlas_2*	rh_Tr-SF_0	*swm_atlas_1*
rh_IT-MT_1	*swm_atlas_1*	rh_PoC-SM_0	*swm_atlas_1*	rh_Tr-SF_1r	*swm_atlas_2*
rh_IT-MT_2	*swm_atlas_1*	rh_PoC-SP_0	*swm_atlas_1*	rh_Tr-Tr_0r	*swm_atlas_2*
rh_LOF-LOF_0i	*swm_atlas_2*	rh_PoC-SP_1	*swm_atlas_1*	UN_RIGHT	DWM

Table S3. Regions and abbreviations of *Desikan-Killiany* atlas (Desikan et al., 2006). The regions are present in both hemispheres.

Region (gyrus)	Abbreviation
Bankssts	Ban
Caudal anterior cingulate	CAC
Caudal middle frontal	CMF
Corpus callosum	COC
Cuneus	Cu
Entorhinal	En
Fusiform	Fu
Inferior parietal	IP
Inferior temporal	IT
Isthmus cingulate	IC
Lateral occipital	LO
Lateral orbito frontal	LOF
Lingual	Li
Medial orbito frontal	MOF
Middle temporal	MT
Parahippocampal	PH
Paracentral	PC
Pars opercularis	Op
Pars orbitalis	Or
Pars triangularis	Tr
Pericalcarine	PeCa
Postcentral	PoC
Posterior cingulate	PoCi
Precentral	PrC
Precuneus	PrCu
Rostral anterior cingulate	RAC
Rostral middle frontal	RMF
Superior frontal	SF
Superior parietal	SP
Superior temporal	ST
Supramarginal	SM
Frontal pole	FRP
Temporal pole	TEM
Transverse temporal	TT
Insula	Ins

Table S4. Number of sub-parcels for each anatomical region (gyrus) for *atlas 5* and *atlas 13*. First column: anatomical parcels from Desikan-Killiany atlas. Second and third columns: number of sub-parcels per hemisphere after applying the parcellation method for *atlas 5*. Fourth and fifth columns: number of sub-parcels per hemisphere for *atlas 13*. The sub-parcels of the *atlas 13* that differ from the *atlas 5* are marked in bold.

Region (gyrus)	# SP lh atlas 5	# SP rh atlas 5	# SP lh atlas 13	# SP rh atlas 13
Bankssts	1	1	**2**	1
Caudal anterior cingulate	1	1	1	1
Caudal middle frontal	3	4	3	4
Cuneus	3	2	**1**	**1**
Entorhinal	2	2	2	**1**
Fusiform	4	3	**5**	3
Inferior parietal	2	3	**6**	**5**
Inferior temporal	4	3	**3**	3
Isthmus cingulate	3	2	3	2
Lateral occipital	2	3	**3**	**2**
Lateral orbito frontal	3	3	**4**	3
Lingual	2	2	**3**	2
Medial orbito frontal	2	2	**3**	2
Middle temporal	3	2	**4**	2
Parahippocampal	1	1	1	1
Paracentral	1	1	1	1
Pars opercularis	1	1	1	1
Pars orbitalis	1	1	**2**	1
Pars triangularis	1	1	1	1
Pericalcarine	1	1	1	1
Postcentral	4	3	**6**	**6**
Posterior cingulate	3	3	3	3
Precentral	7	3	**9**	3
Precuneus	3	2	**4**	**5**
Rostral anterior cingulate	1	1	1	1
Rostral middle frontal	4	2	4	**4**
Superior frontal	4	3	4	**4**
Superior parietal	3	4	**2**	4
Superior temporal	5	4	**6**	4
Supramarginal	4	4	4	4
Frontal pole	1	1	1	1
Temporal pole	1	1	1	1
Transverse temporal	1	1	1	1
Insula	3	2	3	2

Table S5. Number of sub-parcels in common between *atlas 13* and the other atlases generated by the method of parcellation for each anatomical region (gyrus) for a Dice coefficient ≥ 0.6. First column: anatomical parcels from Desikan-Killiany atlas. Second and third columns: number of sub-parcels per hemisphere after applying the parcellation method.

Region (gyrus)	# SP lh	# SP rh
Bankssts	1	1
Caudal anterior cingulate	1	1
Caudal middle frontal	0	3
Cuneus	0	1
Entorhinal	1	1
Fusiform	3	1
Inferior parietal	1	2
Inferior temporal	1	2
Isthmus cingulate	2	1
Lateral occipital	1	1
Lateral orbito frontal	2	3
Lingual	2	1
Medial orbito frontal	2	1
Middle temporal	3	1
Parahippocampal	1	1
Paracentral	1	1
Pars opercularis	0	0
Pars orbitalis	1	0
Pars triangularis	1	1
Pericalcarine	1	1
Postcentral	3	0
Posterior cingulate	1	0
Precentral	2	1
Precuneus	0	2
Rostral anterior cingulate	1	1
Rostral middle frontal	2	1
Superior frontal	2	3
Superior parietal	2	2
Superior temporal	2	1
Supramarginal	1	4
Frontal pole	1	0
Temporal pole	1	1
Transverse temporal	1	1
Insula	3	0

Table S6. Comparison of the number of subdivisions of *Desikan-Killiany (DK)* atlas regions, for the different atlases based on dMRI. First column: the *DK* anatomical parcel. The second and third columns correspond to *atlas 5*, while the fourth and fifth columns correspond to *atlas 13*. The sixth and seventh columns refer to *Lefranc* atlas and the last two columns to the *Brainnetome* atlas. For each atlas, the left and right hemisphere are indicated by *lh* and *rh*, respectively. The numbers that appear in bold with an asterisk in *Brainnetome* represent the parcels that do not perfectly match with *Desikan-Killiany* atlas regions.

Region (gyrus)	lh_atlas 5	rh_atlas 5	lh_atlas 13	rh_atlas 13	lh_Lefranc	rh_Lefranc	lh_BN	rh_BN
Bankssts	1	1	2	1	4	2	2	2
Caudal anterior cingulate	1	1	1	1	4	7	**2***	**2***
Caudal middle frontal	3	4	3	4	3	3	**2***	**2***
Cuneus	3	2	1	1	2	3	**2***	**2***
Entorhinal	2	2	2	1	3	2	**2***	**2***
Fusiform	4	3	5	3	8	7	3	3
Inferior parietal	2	3	6	5	3	3	**3***	**3***
Inferior temporal	4	3	3	3	2	2	7	7
Isthmus cingulate	3	2	3	2	3	6	**2***	**2***
Lateral occipital	2	3	3	2	9	2	6	6
Lateral orbito frontal	3	3	4	3	4	2	**1***	**1***
Lingual	2	2	3	2	2	3	**2***	**2***
Medial orbito frontal	2	2	3	2	2	4	**3***	**3***
Middle temporal	3	2	4	2	6	7	4	4
Parahippocampal	1	1	1	1	3	1	**4***	**4***
Paracentral	1	1	1	1	4	3	2	2
Pars opercularis	1	1	1	1	2	2	**3***	**3***
Pars orbitalis	1	1	2	1	4	3	**1***	**1***
Pars triangularis	1	1	1	1	2	2	**3***	**3***
Pericalcarine	1	1	1	1	3	4	**1***	**1***
Postcentral	4	3	6	6	5	5	4	4
Posterior cingulate	3	3	3	3	3	3	**2***	**2***
Precentral	7	3	9	3	5	5	6	6
Precuneus	3	2	4	5	2	2	4	4
Rostral anterior cingulate	1	1	1	1	2	2	**1***	**1***
Rostral middle frontal	4	2	4	4	5	2	**5***	**5***
Superior frontal	4	3	4	4	5	3	7	7
Superior parietal	3	4	2	4	3	4	5	5
Superior temporal	5	4	6	4	8	3	**4***	**4***
Supramarginal	4	4	4	4	3	5	**3***	**3***
Frontal pole	1	1	1	1	2	3	**1***	**1***
Temporal pole	1	1	1	1	2	2	**1***	**1***
Transverse temporal	1	1	1	1	3	3	**1***	**1***
Insula	3	2	3	2	4	2	6	6

Table S7. Subject datasets used for cross-validation analysis from the 79 subjects of the ARCHI database. Left column: the eight groups of atlas generation and testing datasets. The atlases are named from $cv1$ to $cv8$. Second column: the range of subjects used for the testing datasets. Note that in $cv8$, subject 70 is repeated, to complete 10 subjects. Third column: the range of subjects used for the atlas creation datasets.

# atlas	testing subjects	subjects for the atlas
cv1	1-10	11-79
cv2	11-20	1-10, 21-79
cv3	21-30	1-20, 31-79
cv4	31-40	1-30, 41-79
cv5	41-50	1-40, 51-79
cv6	51-60	1-50, 61-79
cv7	61-70	1-60, 71-79
cv8	70-79	1-69

REFERENCES

Achard, S., Salvador, R., Whitcher, B., Suckling, J., and Bullmore, E. (2006). A resilient, low-frequency, small-world human brain functional network with highly connected association cortical hubs. *Journal of Neuroscience* 26, 63–72

Bassett, D. S., Nelson, B. G., Mueller, B. A., Camchong, J., and Lim, K. O. (2012). Altered resting state complexity in schizophrenia. *NeuroImage* 59, 2196–2207

Bullmore, E. and Sporns, O. (2009). Complex brain networks: graph theoretical analysis of structural and functional systems. *Nature Reviews Neuroscience* 10, 186

Catani, M. (2017). A little man of some importance. *Brain* 140, 3055–3061

Cohen, J. R. and D'Esposito, M. (2016). The segregation and integration of distinct brain networks and their relationship to cognition. *Journal of Neuroscience* 36, 12083–12094

Desikan, R. S., Ségonne, F., Fischl, B., Quinn, B. T., Dickerson, B. C., Blacker, D., et al. (2006). An automated labeling system for subdividing the human cerebral cortex on MRI scans into gyral based regions of interest. *NeuroImage* 31, 968–980

Guevara, M., Román, C., Houenou, J., Duclap, D., Poupon, C., Mangin, J.-F., et al. (2017). Reproducibility of superficial white matter tracts using diffusion-weighted imaging tractography. *NeuroImage* 147, 703–725

Guevara, P., Duclap, D., Poupon, C., Marrakchi-Kacem, L., Fillard, P., Le Bihan, D., et al. (2012). Automatic fiber bundle segmentation in massive tractography datasets using a multi-subject bundle atlas. *NeuroImage* 61, 1083–1099

Humphries, M. D. and Gurney, K. (2008). Network 'small-world-ness': a quantitative method for determining canonical network equivalence. *PLOS ONE* 3, e0002051

Humphries, M. D., Gurney, K., and Prescott, T. J. (2005). The brainstem reticular formation is a small-world, not scale-free, network. *Proceedings of the Royal Society B: Biological Sciences* 273, 503–511

Kohavi, R. et al. (1995). A study of cross-validation and bootstrap for accuracy estimation and model selection. In *IJCAI* (Montreal, Canada), vol. 14, 1137–1145

Román, C., Guevara, M., Valenzuela, R., Figueroa, M., Houenou, J., Duclap, D., et al. (2017). Clustering of Whole-Brain White Matter Short Association Bundles Using HARDI Data. *Frontiers in Neuroinformatics* 11, 73

Rubinov, M. and Sporns, O. (2010). Complex network measures of brain connectivity: uses and interpretations. *NeuroImage* 52, 1059–1069

Telesford, Q. K., Joyce, K. E., Hayasaka, S., Burdette, J. H., and Laurienti, P. J. (2011). The ubiquity of small-world networks. *Brain Connectivity* 1, 367–375

Watts, D. J. and Strogatz, S. H. (1998). Collective dynamics of 'small-world'networks. *Nature* 393, 440
